# Microrheology: From Video Microscopy to Optical Tweezers

**DOI:** 10.3390/mi16080918

**Published:** 2025-08-08

**Authors:** Andrea Jannina Fernandez, Graham M. Gibson, Anna Rył, Manlio Tassieri

**Affiliations:** 1 Division of Biomedical Engineering, James Watt School of Engineering, Advanced Research Centre, University of Glasgow, Glasgow G11 6EW, UK; a.fernandez.1@research.gla.ac.uk (A.J.F.); anna.ryl@p.lodz.pl (A.R.); 2 School of Physics and Astronomy, Advanced Research Centre, University of Glasgow, Glasgow G11 6EW, UK; graham.gibson@glasgow.ac.uk; 3 Department of Chemical and Molecular Engineering, Faculty of Process and Environmental Engineering, Lodz University of Technology, Wolczanska 213, 93-005 Lodz, Poland

**Keywords:** rheology, microrheology, video particle tracking, atomic force microscopy, dynamic light scattering, diffusing wave spectroscopy, magnetic tweezers, optical tweezers

## Abstract

Microrheology, a branch of rheology, focuses on studying the flow and deformation of matter at micron length scales, enabling the characterization of materials using minute sample volumes. This review article explores the principles and advancements of microrheology, covering a range of techniques that infer the viscoelastic properties of soft materials from the motion of embedded tracer particles. Special emphasis is placed on methods employing optical tweezers, which have emerged as a powerful tool in both passive and active microrheology thanks to their exceptional force sensitivity and spatiotemporal resolution. The review also highlights complementary techniques such as video particle tracking, magnetic tweezers, dynamic light scattering, and atomic force microscopy. Applications across biology, materials science, and soft matter research are discussed, emphasizing the growing relevance of particle tracking microrheology and optical tweezers in probing microscale mechanics.

## 1. Introduction

Rheology, derived from the Greek words *rheo* (meaning “flow”) and *logia* (“study of”), is the science that investigates the deformation and flow of matter. It focuses on how materials respond to applied forces, particularly their deformation over time. Central to rheology is the concept of shear flow, which involves the relative motion of parallel planes within a material. This type of flow is crucial for understanding how materials behave under various conditions, including in industrial processes and structural engineering applications, as most materials exhibit a weaker response to shear deformation compared to other forms of deformation.

Microrheology, a branch of rheology, extends these principles to the microscale, enabling the study of material properties on length scales of micrometres or smaller. The roots of microrheology can be traced back to Robert Brown’s 1827 discovery of Brownian motion, where he observed the random, thermally driven motion of pollen particles in water. This observation laid the groundwork for later theoretical advancements by scientists like Albert Einstein [1] and Jean Perrin [2], who helped to establish the foundational principles of microrheology by linking the motion of microscopic particles to the properties of the surrounding medium.

The field of microrheology was formally established by the seminal work of Mason and Weitz [3], who pioneered methods to measure the viscoelastic properties of complex fluids using light-scattering techniques. Their approach involved tracking the Brownian motion of microscopic tracer particles suspended in a fluid and applying the generalized Stokes-Einstein relation to extract the material’s frequency-dependent shear modulus. This innovation provided a powerful means to probe the mechanical properties of soft materials at the microscale. Since then, the field has evolved into two primary branches: “active microrheology”, which involves applying external forces to the tracer particles (e.g., via optical traps or magnetic fields) to probe the material’s response, and “passive microrheology”, which relies on analyzing the spontaneous thermal motion of particles to infer rheological properties.

Microrheology techniques are highly sensitive, capable of detecting forces as small as a few piconewtons and displacements on the nanometre scale, with temporal resolutions commonly reaching microseconds and, in some cases, even stretching down to picoseconds [4]. This sensitivity makes microrheology particularly valuable in studying biological systems where traditional rheological methods are impractical due to the small sample volumes and the complex, often heterogeneous, nature of the materials. However, challenges arise when applying microrheology to non-homogeneous samples, as the assumption of uniform material properties may not hold. In such cases, the local variations experienced by different probe particles can lead to unreliable bulk measurements.

Particle Tracking Microrheology (PTM) is one such technique that has gained prominence due to its ability to monitor the dynamics of biological and soft matter systems at the micron scale. By tracking the movement of individual probe particles, PTM provides a detailed understanding of the viscoelastic properties of complex fluids and biological samples, offering insights that are inaccessible through conventional bulk rheology. Despite its limitations in non-homogeneous materials, PTM remains a powerful tool for studying the mechanical behaviour of small volumes of homogeneous fluids, particularly in biological applications.

**Figure 1:**
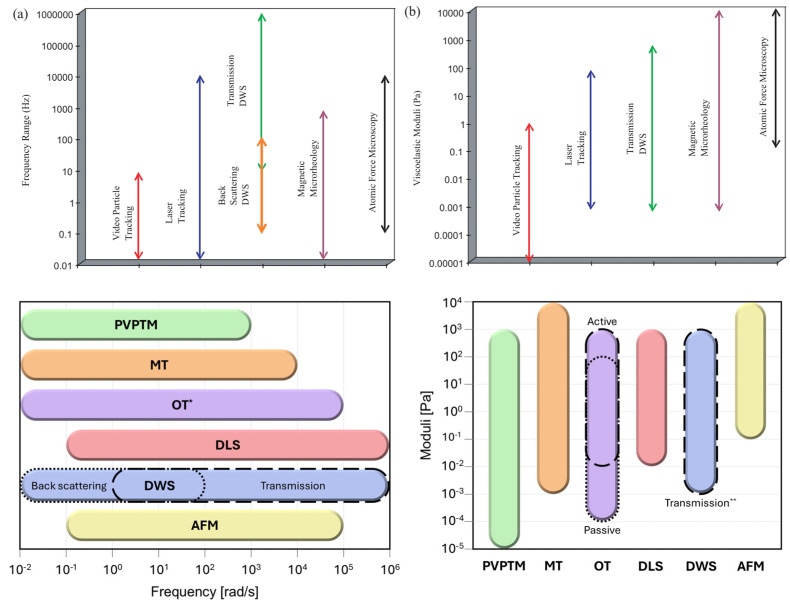
Past (**top** (**a**,**b**), adapted with permission from IOP Publishing, Ltd., reference [5] published in 2005) and current (bottom) accessible ranges of frequencies and magnitudes of viscoelastic moduli obtained from different microrheology methods including passive video particle tracking microrheology (PVPTM) [16,17,18,19,20], magnetic tweezers (MT) [21,22], optical tweezers (OT) [23,24,25,26,27,28], dynamic light scattering (DLS) [29,30,31,32], diffusing wave spectroscopy (DWS) [33,34,35,36,37,38,39], and atomic force microscopy (AFM) [40,41,42,43,44]. * Active and passive microrheology with OT share the same frequency range. ** Experimental results from transmission DWS are more commonly reported compared to back scattering DWS. (Colour-coded in both the diagrams.)

This paper presents an in-depth exploration of Particle Tracking Microrheology, discussing its principles, methodologies, and applications in modern scientific research. However, this review article cannot be exhaustive; therefore, we refer the reader to some of the most established review articles on the subject [5,6,7,8,9,10,11], as well as to more recent contributions [12,13,14,15]. Notably, when comparing earlier work with more recent studies (see Figure 1), one observes a substantial expansion in the sensitivity of most microrheology techniques—both in terms of accessible frequency ranges and the magnitudes of measurable viscoelastic moduli of complex materials. This improvement is primarily due to technological advancements in instrumentation since the initial development of these techniques. Figure 1 presents an up-to-date estimation of these ranges, as reported in recent publications.

A summary of the main advantages and limitations of each technique is provided in Table 1 for quick reference and comparison.

## 2. Theoretical Background

### 2.1. Rheology in Simple Shear Flow

In linear rheology, shear deformation is the most relevant type of deformation as it effectively describes the laminar flow behaviour of fluids. Moreover, many materials exhibit weaker mechanical resistance under shear compared to other types of deformation, such as compressional or torsional stresses. This sensitivity to shear makes it particularly valuable in considerations of material performance, safety, and the design and operation of rheological instrumentation.

**Figure 2:**
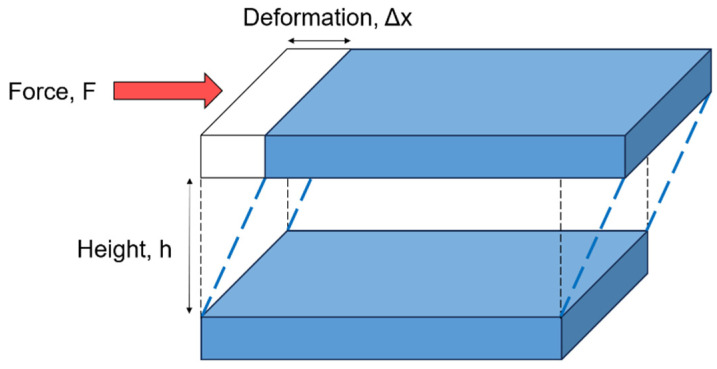
A schematic of the two-plate model for shear deformation.

Shear deformation can be illustrated using the two-plate model, depicted in Figure 2. In this model, a material is placed between two parallel plates separated by a fixed distance. The flow of the material is induced by a shear stress $\left(\sigma \right)$, which is defined as the ratio of the applied tangential force $\left(F\right)$ to the contact area $\left(A\right)\;$ of the upper plate in contact with the material, assuming the lower plate remains stationary. Shear stress is expressed in Pascals (Pa) according to the International System of Units (SI). The resulting deformation is described by the shear strain $\left(\gamma \right)$, which is defined as the ratio of the relative displacement $\left(∆x\right)$ to the separation height $\left(h\right)$ of the two plates. This yields a dimensionless quantity. Similarly, shear flow can be characterized by the rate of deformation, referred to as the shear rate or strain rate, which is the time derivative of the shear strain $\left(\dot{\gamma }=d\gamma /dt\right)$. The shear rate is expressed in the International System of Units (SI) as reciprocal seconds (s^−1^).

All real materials exhibit mechanical properties that lie between two quasi-ideal extremes: (1) a perfectly elastic solid and (2) a purely viscous fluid. According to Hooke’s law, a perfectly elastic solid subjected to small deformations experiences a stress that is directly proportional to the strain, regardless of the strain rate. This behaviour reflects the material’s ability to store deformation energy and recover its original shape upon the removal of stress. In the context of shear deformation, the relationship for a perfectly elastic solid is expressed as:

(1) $$\sigma \left(t\right)=G\gamma \left(t\;\right)$$

where G is the time-independent shear elastic modulus, analogous to the Young’s modulus $\left(E\right)$ for shear deformation, with the unit of Pascal (Pa).

Conversely, for a purely viscous fluid, stress is directly proportional to the strain rate and independent of strain, as described by Newton’s law of viscosity:

(2) $$\sigma \left(t\right)=\eta \frac{d\gamma \left(t\right)}{dt}$$

where η  is the Newtonian viscosity of the fluid, a time-independent parameter with units of Pascal-seconds (Pa⋅s). In reality, both G and η can be considered time-independent constants only within a finite stress and strain range, which defines the material’s Linear Viscoelastic (LVE) regime. To fully capture the viscoelastic behaviour of real materials, more complex constitutive equations are necessary.

In simple shear, the constitutive equation for linear viscoelasticity is based on the principle that the effects of sequential changes in strain are additive, and it can be expressed as [45]:

(3) $$\sigma \left(t\right)=\int_{-\infty }^{t}G\left(t-t^{\prime }\right)\dot{\gamma }\left(t^{\prime }\right)dt^{\prime }$$

where $G\left(t\right)\;$ is the time-dependent shear relaxation modulus of the material.

From Equation (3), it is an easy step to express the LVE properties of a generic material in terms of its shear complex modulus $G^{*}\left(\omega \right)$, whose real and imaginary parts describe the elastic and viscous nature of the material, respectively:

(4) $$G^{*}\left(\omega \right)=G^{\prime }\left(\omega \right)+iG^{\prime \prime }\left(\omega \right)$$

where ω is the angular frequency, i is the imaginary unit ($i^{2}=-1\;$), and $G^{\prime }\left(\omega \right)$ and $G^{\prime \prime }\left(\omega \right)$ are the frequency-dependent storage (elastic) and loss (viscous) moduli of the material, respectively. The complex shear modulus can be defined as the ratio of the Fourier transform of the stress to that of strain, or equivalently as the Fourier transform of the time derivative of the shear relaxation modulus $G\left(t\right)$ [45]:

(5) $$G^{*}\left(\omega \right)=\frac{\hat{\sigma }\left(\omega \right)}{\hat{\gamma }\left(\omega \right)}=FT\left[\frac{dG\left(t\right)}{dt}\right]=i\omega \hat{G}\left(\omega \right)$$

An alternative way to describe the viscoelastic nature of a material is by means of its dynamic compliance $J^{*}\left(\omega \right)$, which, in the frequency domain, can be defined as the inverse of $G^{*}\left(\omega \right)$:

(6) $$J^{*}\left(\omega \right)=\frac{\hat{\gamma }\left(\omega \right)}{\hat{\sigma }\left(\omega \right)}=i\omega \hat{J}\left(\omega \right)$$

where $\hat{J}\left(\omega \right)$ is the Fourier transform of the time-dependent creep compliance $J\left(t\right)$, which has a unit of Pa^−1^ and is defined as:

(7) $$J\left(t\right)=\frac{\gamma \left(t\right)}{\sigma_{0}}$$

where $\sigma_{0}$ is the amplitude of a constant stress applied at time equal zero.

Conventionally, to measure $G^{*}\left(\omega \right)$ of a generic material over a finite range of frequencies, an oscillatory stress $\sigma \left(\omega ,t\right)=\sigma_{0}\sin\left(\omega t\right)$ is applied, where $\sigma_{0}$ is its amplitude. The resulting oscillatory strain $\gamma \left(\omega ,t\right)=\gamma_{0}\sin\left(\omega t-\varphi \left(\omega \right)\right)$ is then measured; where $\gamma_{0}$ is the amplitude of the strain and $\varphi \left(\omega \right)$ is the phase shift between the stress and strain. In follows that the complex shear modulus can thus be further explicated as:

(8) $$G^{*}\left(\omega \right)=\frac{\sigma_{0}}{\gamma_{0}\left(\omega \right)}\cos\left(\varphi \left(\omega \right)\right)+i\frac{\sigma_{0}}{\gamma_{0}\left(\omega \right)}\sin\left(\varphi \left(\omega \right)\right)$$

from which the expressions of $G^{\prime }\left(\omega \right)$ and $G^{\prime \prime }\left(\omega \right)$ in Equation (4) are revealed. Interestingly, all materials experience a phase shift $0\leq \varphi \left(\omega \right)\leq \frac{\pi }{2}$ when subjected to oscillating stress. Depending on the frequency range explored, these materials can exhibit asymptotic behaviours characteristic of either perfectly elastic solids or purely viscous fluids. Specifically, for frequencies where the phase shift $\varphi \left(\omega \right)$ approaches 0, the material behaves like an elastic solid; whereas, for frequencies where $\varphi \left(\omega \right)$ approaches $\frac{\pi }{2}$, the material behaves like a Newtonian fluid [46].

### 2.2. Passive and Active Microrheology

Most microrheology techniques involve the suspension of micron- or nano-sized spherical particles, also known as probe or tracer particles, into the fluid under investigation. As previously mentioned, existing microrheology techniques are defined as either ‘passive’ or ‘active’, depending on whether the motion of these particles is caused by the thermal energy via random collisions with fluid molecules (i.e., Brownian motion) or induced by an external force. In either case, the viscoelastic properties of the suspending fluid are determined by solving the relationship between the driving force and the trajectory of the tracer particles, which are monitored over time. This approach was pioneered by Mason and Weitz [3], who established the field of microrheology by correlating the mean squared displacement (MSD) of diffusing particles to the complex shear modulus $G^{*}\left(\omega \right)$ of the suspending fluid, as elucidated hereafter.

#### 2.2.1. Passive Microrheology

The mean squared displacement (MSD), $\langle \Delta r^{2}\left(\tau \right)\rangle \equiv \langle {\left[\vec{r}\left(t_{0}+\tau \right)-\vec{r}\left(t_{0}\right)\right]}^{2}\rangle$, of a particle is a function of its position and depends only on the lag time $\tau =\left(t-t_{0}\right)$, where $t_{0}$ is a generic initial observation time. For a Newtonian fluid, where the viscosity η remains constant, the MSD is directly proportional to the lag time:

(9) $$\langle \Delta r^{2}\left(\tau \right)\rangle =2dD\tau$$

where d is the number of observed dimensions (d=3 for three-dimensional trajectories) and D is the diffusion coefficient, defined by the Stokes-Einstein relation:

(10) $$D=\frac{k_{B}T}{6\pi a\eta }$$

where $k_{B}$ is the Boltzmann constant, T is the absolute temperature, and a is the radius of the particle. By combining Equations (9) and (10), the viscosity of the suspending fluid can easily be determined once the particles size is known:

(11) $$\langle \Delta r^{2}\left(\tau \right)\rangle =\frac{dk_{B}T}{3\pi a\eta }\tau$$

In the case of non-Newtonian fluids, the viscoelastic properties of a complex fluid have been linked to the MSD of a suspended particle by means of a generalized Langevin equation (GLE), which describes the thermally driven motion of the particle:

(12) $$m\vec{a}\left(t\right)={\vec{f}}_{R}\left(t\right)-\int_{-\infty }^{t}\zeta \left(t-t^{\prime }\right)\vec{v}\left(t^{\prime }\right)dt^{\prime }$$

where m, $\vec{v}\left(t\right)$, and $\vec{a}\left(t\right)$ are the mass, velocity, and acceleration of the particle, respectively. The term ${\vec{f}}_{R}\left(t\right)$ describes random forces acting on the particle, due to both direct forces between particles and stochastic thermal forces. Furthermore, $\zeta \left(t\right)$ is a time-dependent memory function that represents the viscous damping force. By following the assumption made by Mason and Weitz [3] on $\tilde{\zeta }\left(s\right)$ being proportional to the Laplace-transformed viscosity of the fluid $\tilde{\eta }\left(s\right)$:

(13) $$\tilde{\zeta }\left(s\right)=6\pi a\tilde{\eta }\left(s\right)$$

Equation (12) can be solved for the materials’ shear complex modulus in terms of the MSD, giving the Generalized Stokes Einstein Relation (GSER):

(14) $$G^{*}\left(\omega \right)=s\;\tilde{\eta }\left(s\right){|}_{s=i\omega }=\frac{1}{6\pi a}\left[\frac{6k_{B}T}{i\omega \langle \Delta {\hat{r}}^{2}\left(\omega \right)\rangle }+m\omega^{2}\right]$$

where $\langle \Delta {\hat{r}}^{2}\left(\omega \right)\rangle$ is the Fourier transform of the MSD. The second term in the brackets, representing an inertial effect, is commonly neglected, as it becomes significant only at very high frequencies, typically on the order of MHz for micron-sized particles. Levine and Lubensky [47] provided further theoretical insights into the validity of the generalized Stokes-Einstein relation, introducing a dimensionless, frequency-dependent term to assess the significance of fluid inertia:

(15) $$\beta_{F}\left(\omega \right)=\frac{4a^{2}\omega^{2}\rho_{F}}{G{\left(\omega \right)\pi }^{2}}$$

where $\rho_{F}$ is the density of the fluid. Likewise, they also provided a term for the contribution of particle inertia:

(16) $$\beta_{b}\left(\omega \right)=\frac{2a^{2}\omega^{2}\rho_{b}}{9G\left(\omega \right)}$$

where $\rho_{b}$ is the density of the tracer particle. The GSER is said to be valid for the frequency range where the inertial effects of both the fluid and the particle are negligible. This occurs when $\omega <\omega^{*}$, where $\beta_{F}\left(\omega^{*}\right)\;\sim \;\beta_{b}\left(\omega^{*}\right)\;\sim \;1$, translating to an upper frequency limit on the order of MHz. Furthermore, the contribution of the longitudinal compressional mode of the fluid must also be negligible to ensure that microrheology measurements have good agreement with bulk rheology [47].

#### 2.2.2. Active Microrheology

Active microrheology methods apply stresses significantly larger than the thermal fluctuations within the fluid, enabling the characterization of stiffer materials and the observation of their nonlinear and nonequilibrium responses [48]. When a driving force $\vec{F}\left(t\right)$ is applied to the probe particles, the generalized Langevin equation provided in Equation (12) becomes:

(17) $$m\vec{a}\left(t\right)={\vec{f}}_{R}\left(t\right)+\vec{F}\left(t\right)-\int_{0}^{t}\zeta \left(t-t^{\prime }\right)\vec{v}\left(t^{\prime }\right)dt^{\prime }$$

As discussed in later sections, the force term $\vec{F}\left(t\right)$ varies depending on the method of application, and therefore, the analytical solution of Equation (17) will be tailored to the specific experimental procedure employed.

### 2.3. Image Analysis

To track the motion of particles, two approaches are generally used. The first involves the direct monitoring of particles through video microscopy, while the other relies on the detection of light scattered from tracer particles, offering a more indirect approach.

#### 2.3.1. Video Microscopy

Some microrheology techniques such as passive video particle tracking, magnetic and optical tweezers rely on video microscopy to directly observe and record the motion of probe particles. Bright-field, fluorescence, and confocal microscopy can be used for capturing videos depending on the nature of the sample, size of tracer particles, and the required spatial resolution. The position of the particle versus time is then determined through image analysis. This can be performed using a number of open-access software that have been written in different programming languages [49].

Particle tracking was first demonstrated by Crocker and Grier [50], who developed an image processing algorithm using the programming language IDL (Interactive Data Language). This method is versatile, applicable to a wide range of colloidal systems, and supports both single-particle and multi-particle tracking. Their approach involves five key stages: (i) background and noise removal, (ii) particle localization, (iii) refinement of particle position estimates, (iv) noise discrimination, and (v) linking particle positions to form trajectories.

Background subtraction aids in correcting contrast variations that may arise due to uneven illumination of the sample, which in turn can affect the easy recognition of spheres. The background image can be modelled by a boxcar average over a region of 2w+1, where w is an integer in pixels larger than a sphere’s apparent radius, but smaller than the interparticle separation distance:

(18) $$A_{w}\left(x,y\right)=\frac{1}{{\left(2w+1\right)}^{2}}\sum_{i,j=-w}^{w}A\left(x+i,y+j\right)$$

Meanwhile, noise that arises from digitization can be suppressed using a Gaussian revolution of pixel half width $\lambda_{n}=1$:

(19) $$A_{\lambda_{n}}\left(x,y\right)=\frac{\sum_{i,j=-w}^{w}A\left(x+i,y+j\right)\exp\left(-\frac{i^{2}+j^{2}}{4\lambda_{n}^{2}}\right)}{B}$$

where B is a normalization factor equal to:

(20) $$B={\left[\sum_{i=-w}^{w}\exp\left(-\frac{i^{2}}{4\lambda_{n}^{2}}\right)\right]}^{2}$$

The ideal image is best estimated by obtaining the difference between $A_{w}\left(x,y\right)$ and $A_{\lambda_{n}}\left(x,y\right)$. Because both Equations (18) and (19) are taken as convolutions of the image $A\left(x,y\right)$ over the same region 2w+1, they can be computed in a single step through the equation:

(21) $$K\left(i,j\right)=\frac{1}{K_{0}}\left[\frac{1}{B}\exp\left(-\frac{i^{2}+j^{2}}{4\lambda_{n}^{2}}\right)-\frac{1}{{\left(2w+1\right)}^{2}}\right]$$

where $K_{0}$ is a normalization constant equal to [50]:

(22) $$K_{0}=\frac{1}{B}{\left[\sum_{i=-w}^{w}\exp\left(-\frac{i^{2}}{2\lambda_{n}^{2}}\right)\right]}^{2}-\frac{B}{{\left(2w+1\right)}^{2}}$$

Particle locations are then taken as the local brightness maxima within the image. Initially, candidate locations for particle centroids are identified if a given pixel $A\left(x,y\right)$ is brighter than any other pixel within a distance of w pixels. Furthermore, to reduce error, candidate pixels can be given an additional criterion of being within the top 30% of brightest pixels within the entire image. The identification process is facilitated through grayscale dilation, where a pixel is set to the maximum value within the area w. The pixel in the original image with the same value in the dilated image is then regarded as a candidate [50].

After identifying candidate locations at $\left(x,y\right)$, the actual centroids or the geometric centres $\left(x_{0},y_{0}\right)$ of the sphere can be calculated by determining the offset $\left(\epsilon_{x},\epsilon_{y}\right)$ between the two coordinates:

(23) $$\left(\begin{array}{c} \epsilon_{x}\\ \epsilon_{y} \end{array}\right)=\frac{1}{m_{0}}\sum_{i^{2}+j^{2}\leq w^{2}}\left(\begin{array}{c} i\\ j \end{array}\right)A\left(x+i,y+j\right)$$

where $m_{0}$ is the total summation of the brightness within the sphere, given by:

(24) $$m_{0}=\sum_{i^{2}+j^{2}\leq w^{2}}A\left(x+i,y+j\right)$$

The exact centroid of the sphere is hence located at $\left(x_{0},y_{0}\right)=\left(x_{0}+\epsilon_{x},\;y_{0}+\epsilon_{y}\right)$ [50]. Following this, the eccentricity e of the tracked objects can be calculated as an additional identifier to disregard non-spherical objects. These objects may be in the form of aggregated particles, impurities such as dust, and imperfections in the optical system. Perfectly circular particles have an eccentricity of e=0, whereas perfect lines would have an eccentricity of e=1 [51].

Noise can further be discriminated by calculating the moment of brightness distribution given by Equation (24) replacing $\left(x,y\right)$ with the coordinates of the centroid of the particle $\left(x_{0},y_{0}\right)$. A second moment $m_{2}$ can also be obtained:

(25) $$m_{2}=\frac{1}{m_{0}}\sum_{i^{2}+j^{2}\leq w^{2}}\left(i^{2}+j^{2}\right)A\left(x_{0}+i,y_{0}+j\right)$$

**Figure 3:**
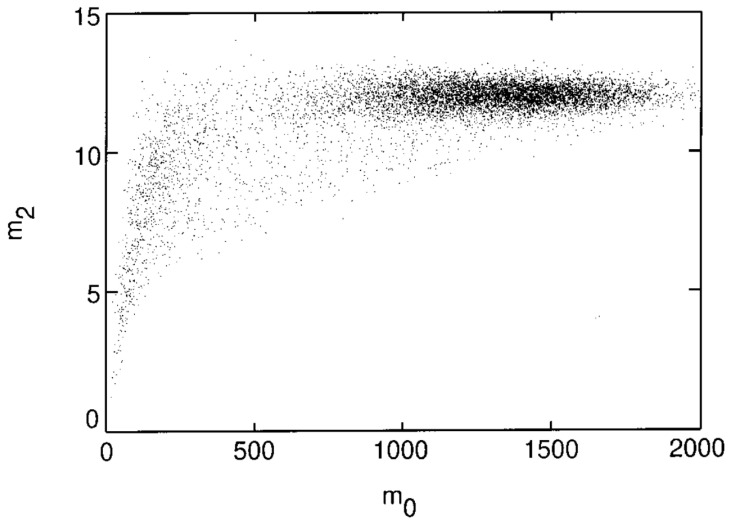
Clustering of colloidal images in the ${(m_{0},m}_{2})$ plane. 15,000 images of σ = 0.325 µm radius spheres. Reprinted with permission from Ref. [50]. Copyright 1996 Elsevier.

**Figure 4:**
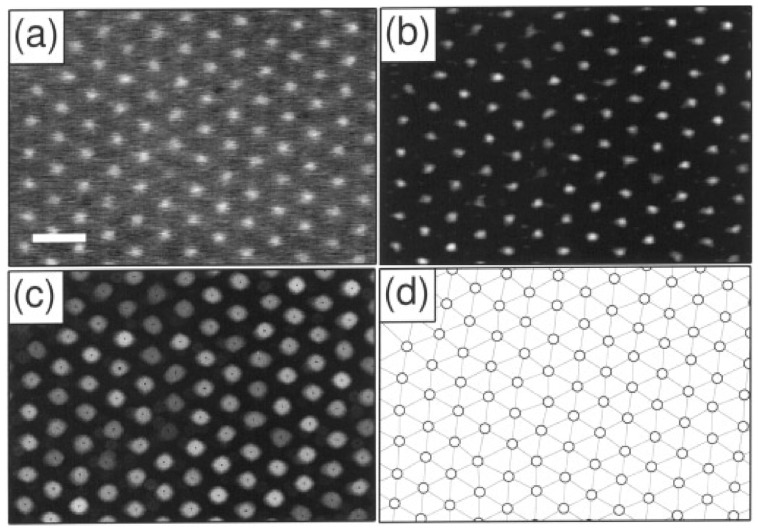
Stages of image processing. (**a**) Detail of a video micrograph of the (111) plane of a face-centred cubic colloidal crystal. The radius of each polystyrene sulfonate sphere is σ = 0.163 µm. The scale bar indicates 2 µm. (**b**) The same image filtered with the convolution kernel in Equation (21). (**c**) Grey-scale dilation of the image in (**b**). Dark spots represent the initial estimates for particle locations based on the neighbourhood maximum algorithm. (**d**) Final particle location estimates. The lines connecting sites constitute the network of nearest-neighbour bonds computed as a Delaunay triangulation (Preparata, F. P., and Shamos, M. I., “Computational Geometry.” Springer-Verlag, New York, 1985.) Such a network is useful as the basis of many measurements of local ordering. Reprinted with permission from Ref. [50]. Copyright 1996 Elsevier.

These moments help ascertain the size of the identified particles and can thus be used to determine their relative location from the focal plane. As seen in Figure 3, the colloidal spheres tend to distribute into broad clusters within the ${(m_{0},m}_{2})$ plane, which also arises from the variation in distances in the direction normal to the imaging plane. Notably, a decrease in $m_{2}$ at low $m_{0}$ reflects the fact that dimmer objects are often out of focus, smaller, or represent noise, and therefore exhibit lower spatial variance in intensity. Statistical cluster analysis is used to filter out the noise and to distinguish different particle types from one another, which is useful in the case of bi- and poly-disperse suspensions. The first four stages of the tracking implementation can be seen in Figure 4 [50].

Finally, particle trajectories are formed by linking the locations of particles in successive image frames. For monodisperse colloidal suspensions where particles are indistinguishable, the likelihood that one particle corresponds to another in a previous frame is estimated through their proximity in the two images. Hence, to find the most likely set of particle locations that evolved from those in a previous image, a probability distribution function describing Brownian motion is considered. For a single particle with a diffusion coefficient D, the probability that it will diffuse at a distance δ in time τ is:

(26) $$P\left(\delta |\tau \right)=\frac{1}{4\pi D\tau }\exp\left(-\frac{\delta^{2}}{4D\tau }\right)$$

When the system contains N identical, non-interacting particles such as in the case of multiple particle tracking, the probability distribution becomes the product of N single particle distributions:

(27) $$P\left(\left\{\delta_{i}\right\}|\tau \right)={\left(\frac{1}{4\pi D\tau }\right)}^{N}\exp\left(-\sum_{i=1}^{N}\frac{\delta_{i}^{2}}{4D\tau }\right)$$

Using Equation (27) to link particle trajectories means finding bonds between two successive frames that maximizes the probability $P\left(\left\{\delta_{i}\right\}|\tau \right)$, or minimizes the squared distances travelled by all particles $\sum_{i=1}^{N}\delta_{i}^{2}$. To limit the number of possible combinations that are considered and lessen the computational demand for the process, a length L can be assigned such that bonds longer than this length will be disregarded. This is similar to truncating the probability distribution $P\left(\delta |\tau \right)$ at δ=L [50]. This simplification can be performed as long as the interparticle separation distance d is much larger than the distance travelled by particles, else particles that are close to one another may swap positions once trajectories are linked. To circumvent this, the particle concentration in a sample can be lowered [51]. Ideally, L should also be chosen such that $\delta <L<\frac{d}{2}$ [50].

Particles may appear or disappear from the field of view of the microscope in between frames, causing some bonds to be missing when linking particle trajectories. To ensure that Equation (27) can be evaluated despite this, the missing bonds are assigned the length $\delta_{i}=L$. The affected particles are also labelled as missing at certain time steps, and their last known locations are stored in case a particle would appear close to it to resume the trajectory linking [50]. A memory number $n_{\mathrm{mem}}$ can be assigned, indicating the maximum number of consecutive frames a particle can be missing before it is treated as a new particle upon reappearing. Similarly, objects that only appear for a single frame and may contribute to erroneous data can be eliminated by specifying $n_{\min}$, or the minimum number of frames a particle must be present in the video [51].

To address challenges in particle tracking in three dimensions (3D), optical techniques have advanced significantly, particularly through stereoscopic imaging and structured illumination. Structured illumination using a digital projector, as demonstrated by Dam et al. [52], offers dynamic 3D visualization, allowing for real-time adjustments to illumination patterns for enhanced tracking and manipulation. This approach employs colour-coded illumination and stereo cameras to map particles’ 3D coordinates accurately while maintaining flexibility in illumination configurations. Alternatively, stereoscopic microscopy, as described by Bowman et al. [53] and Lee et al. [54], utilizes dual-view imaging systems to resolve axial and lateral positions of particles with high precision. Their technique employs Fourier-domain optical filters to generate stereoscopic pairs, achieving axial resolutions down to 3 nm at 340 Hz frame rates. Both methods represent crucial innovations in overcoming defocusing and ghosting issues in conventional tracking, providing precise, scalable solutions for studying microscale dynamics in complex systems.

#### 2.3.2. Light Scattering or Non-Direct Tracking

Particle locations can also be tracked without the use of video microscopy. Microrheology techniques such as Dynamic Light Scattering (DLS) and Diffusing Wave Spectroscopy (DWS) rely instead on the scattering and detection of light that hits the sample under investigation.

Light interacts with matter through four major ways, namely absorption, emission, transmission, and reflection. During the transmission of light through a medium, light scattering may also occur [55]. During this process, the scattering centre receives incident light, which has a given frequency and propagation vector. The scattering centre then emits light with a changed propagation vector, either with or without the accompaniment of a changed frequency. The first case where the scattered light only has a change in the propagation vector is referred to as elastic scattering. Meanwhile, the second case with a change in both propagation vector and frequency is called inelastic scattering [56].

Elastic scattering may take place through two possible mechanisms. First, the incident light may only simply have a change in direction, similar to the reflection of light from a smooth or rough surface. Second, and more relevant to present context, the incident photons may be absorbed by a molecular process then reemitted without noticeable changes in frequency. This process is referred to as Rayleigh or Mie scattering depending on the scattering centre involved [56].

Named after Lord Rayleigh, who first theorized the process to explain the colour of the sky, Rayleigh scattering occurs when the particles scattering the incident light are much smaller than the wavelength of the incident light. Typically, the diameter of the scattering centre should be less than $\frac{\lambda }{10}$, where λ is the wavelength of the incident light [57]. The oscillating electric field of the incident light wave excites bound electrons within the atom or molecule, causing them to oscillate at the same frequency as the incident wave and release electromagnetic radiation [58]. For unpolarized incident light with intensity $I_{0}$, the Rayleigh scattered intensity $I_{s}$ is given by:

(28) $$I_{s}=I_{o}\frac{8\pi^{4}Na^{6}}{\lambda^{4}r^{2}}\left|\frac{m^{2}-1}{m^{2}+1}\right|\left(1+\cos^{2}\theta \right)$$

where N is the number of particles, a is the radius of the particles, θ is the scattering angle, r is the distance of the observation point from the scattering centre, and m is the ratio of the refractive indices of the scattering centre to the medium (i.e., $m=\frac{n_{1}}{n_{0}}$). The $\lambda^{-4}$ dependence explains why shorter wavelengths of light scatter more compared to longer wavelengths [56].

Meanwhile, Mie scattering is named after Mie, who developed a general theory for the scattering of electromagnetic waves by spherical particles of any size. Mie scattering is particularly observed when the scattering centres are larger than those involved in Rayleigh scattering (i.e., diameters greater than $\frac{\lambda }{10}$). Mie scattering has two features that become more pronounced when the scattering centres become much larger than λ. First, with increasing particle size, more light scatters in the forward direction compared to the back direction. Second, the scattering of lower wavelengths becomes less dominant until all wavelengths are scattered equally [58].

Once light is scattered by atoms or molecules in a sample, the emitted light waves will undergo either destructive or constructive interference. A detectable signal is only formed in the latter case, since out-of-phase waves in the former cancel each other out. The signal intensity is recorded by a detector [59] and often processed in real time to construct a correlation function between the intensity fluctuations of the scattered light and the dynamics of the scattering centres, as explained below.

## 3. Most Popular Microrheology Techniques

### 3.1. Passive Video Particle Tracking Microrheology

Passive video particle tracking microrheology (PVPTM) has revolutionized the study of mechanical properties in complex fluids and soft materials. By analyzing the random thermal motion of microscopic tracer particles, PVPTM enables the measurement of viscoelastic properties without requiring external forces, making it a quasi-non-invasive and versatile tool across multiple scientific domains.

**Figure 5:**
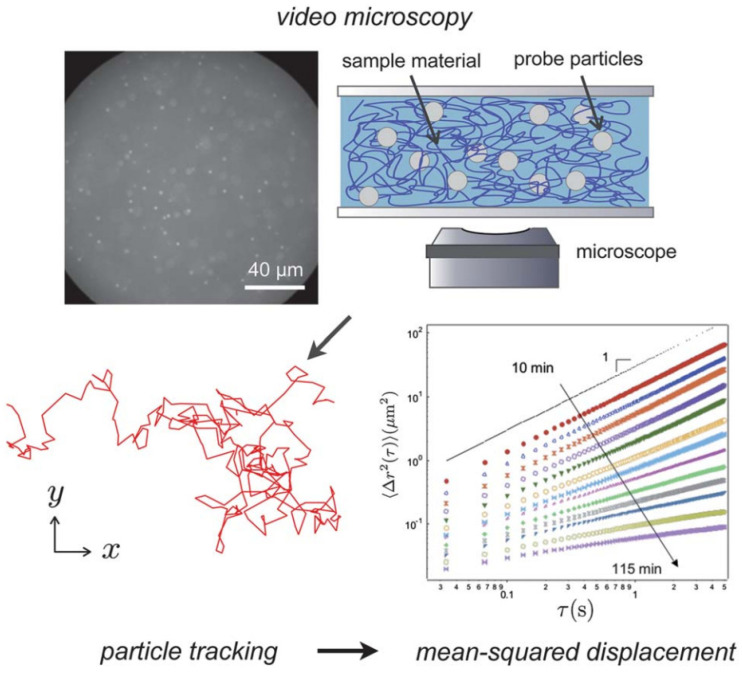
Multiple particle tracking microrheology measures random thermal motion of colloidal probe particles embedded in a soft material. Video microscopy images are processed to calculate individual trajectories. The ensemble average of the tracer mean-squared displacements is a measure of the material rheology by the Generalized Stokes–Einstein Relation. In the case shown, the material is gelling with time, leading to a series of curves ranging from a viscous liquid $\left(\langle \Delta r^{2}\left(t\right)\rangle \;\sim \;t\right)$ to an elastic gel $\left(\langle \Delta r^{2}\left(t\right)\rangle \;\sim \;\mathrm{constant}\right)$. The mean-squared displacement plot is reprinted with permission from T. H. Larsen and E. M. Furst, Phys. Rev. Lett., 2008, 100, 146001. Reprinted with permission from Ref. [10]. Copyright 2012 Royal Society of Chemistry.

In biological systems, PVPTM has been instrumental in uncovering the viscoelastic properties of living cells. Studies involving the tracking particles embedded in or attached to the cytoplasm have revealed that cellular interiors exhibit complex viscoelastic behaviours driven by the cytoskeleton and intracellular organelles. These findings have provided critical insights into how mechanical properties influence key cellular functions such as division, migration, and mechanotransduction [11,60]. The method has also been applied to the extracellular matrix and biofilms, helping researchers understand their mechanical stability and how they support biological processes or resist external stress. Such studies have implications for tissue engineering and therapeutic developments [61]. For instance, PVPTM can be used to evaluate drug efficacy by correlating the dynamics of probes to structural changes occurring in the cell over time. The live response of murine fibroblasts to nocodazole, which causes microtubule depolymerization, has been investigated. At the start of tracking, probes were undergoing ballistic motion due to cells crawling on the substrate. Once microtubule depolymerization occurred, cells ceased to crawl, and it was observed that probe particles appeared elastically caged, and the percentage of caged probes were monitored over time [62]. A more recent application of PVPTM explored the viscoelastic properties of glioblastoma cells, focusing on their mechanical heterogeneity and its role in cellular invasiveness. This study combined particle tracking with advanced analytical techniques to assess how the mechanical environment within tumour cells correlates with their aggressive behaviour, providing valuable insights for understanding cancer progression and developing potential therapeutic strategies [63]. Apart from biological systems, PVPTM can also help in the characterization of hydrogels as schematically shown in Figure 5. Hydrogels are explored as materials to facilitate drug delivery, tissue engineering, and wound healing, and rheological assessment is critical in these applications where material stiffness can serve as a biophysical cue to surrounding or seeded cells [10].

PVPTM has also proven valuable in food science, where it is used to investigate the rheological properties of gels, emulsions, and other complex food matrices. Tracer particles allow researchers to monitor how structural and mechanical properties change during food processing and storage, offering insights into optimizing texture and stability. This understanding helps ensure product quality and consistency, which are critical for industrial applications [64].

In the field of materials science, PVPTM has been extensively applied to polymer and protein solutions. By tracking particle motion in these systems, researchers have characterized molecular interactions, gelation dynamics, and phase transitions, advancing the design of new materials. For instance, microrheological studies have elucidated the behaviour of protein aggregates, which is important for both industrial formulations and biomedical applications [65]. Similarly, PVPTM has enhanced our understanding of polymer dynamics, aiding in the development of materials with tailored mechanical properties [66].

Recent methodological advancements have further expanded the capabilities of PVPTM. Techniques like multiple-particle tracking and two-point microrheology allow for detailed analyses of spatial heterogeneity and anisotropic properties within samples [51,60]. Additionally, integrating PVPTM with cutting-edge imaging technologies such as confocal and super-resolution microscopy has improved spatial resolution and enabled three-dimensional tracking, particularly useful for thick or optically dense samples [66].

However, challenges remain in interpreting data from heterogeneous or complex systems, where factors like particle size, surface chemistry, and interactions with the medium can affect measurements. Efforts are ongoing to standardize protocols and improve analytical frameworks, ensuring the reproducibility and reliability of microrheological data [67]. For living cells, several approaches have already been developed to better understand underlying cellular processes and mechanics from particle tracking experiments [68]. Looking ahead, combining PVPTM with computational modelling and machine learning offers exciting possibilities for deeper insights into micromechanical properties and the design of materials with targeted functionalities [66,69].

Overall, PVPTM has emerged as a transformative tool, bridging diverse fields such as biology, food science, and materials engineering. Its ability to non-invasively probe the micro-scale mechanics of complex systems has not only enhanced fundamental understanding but also driven innovation in applications ranging from cell biology to industrial formulation. As methodological advancements continue, PVPTM’s role in scientific discovery and practical applications is set to grow even further.

### 3.2. Magnetic Tweezers

Magnetic Tweezers (MT) are experimental setups based on electromagnetic apparatuses that generate magnetic fields to manipulate, in 3D, either ferromagnetic or superparamagnetic micron-sized beads. MT can be considered the first active setup used in microrheology, with early applications dating back to the 1920s with the work performed by the botanist Heilbronn [70]. MT became popular in biophysical studies after the work by Crick and Hughes on the physical properties of cytoplasm [71].

The working principles of MT are based on the use of electromagnetic coils to generate a magnetic field $\vec{B}$ that interacts with the (induced or permanent) magnetic moment $\vec{m}$ of a probe particle. This interaction generates a magnetic force ${\vec{F}}_{m}$ on the particle:

(29) $${\vec{F}}_{m}=\nabla \left(\vec{m}\cdot \vec{B}\right)$$

with

(30) $$\vec{m}\left(t\right)=\frac{4\pi a^{3}}{3}\rho \left[{\vec{m}}_{0}+\frac{\chi }{\rho \mu_{0}}\vec{B}\left(t\right)\right]$$

where ${\vec{m}}_{0}$ denotes the initial magnetization of the particle, ρ its specific density, χ the magnetic susceptibility of the probe, and $\mu_{0}$ the permeability of the vacuum. In practice, given that $\left|\vec{B}\left(t\right)\right|\propto I\left(t\right)$ one can generate a time-dependent magnetic field $\vec{B}\left(t\right)$ by controlling the electric current $I\left(t\right)$ in the electromagnetic coils. Therefore, the generated magnetic force ${\vec{F}}_{m}\left(t\right)$ could have the desired temporal form such as a sinusoidal function or creep function, as commonly used in oscillatory bulk experiments.

In oscillatory measurements, a sinusoidal force is applied to the probe particle. Once the probe displacement and phase shift are measured, $G^{\prime }\left(\omega \right)$ and $G^{\prime \prime }\left(\omega \right)$ can be determined through the following equations:

(31) $$G^{\prime }\left(\omega \right)=\frac{f_{0}}{6\pi ax\left(\omega \right)}\cos\varphi \left(\omega \right)$$

(32) $$G^{\prime \prime }\left(\omega \right)=\frac{f_{0}}{6\pi ax\left(\omega \right)}\sin\varphi \left(\omega \right)$$

where $f_{0}$ is the amplitude of the applied force, $x\left(\omega \right)$ is the frequency-dependent displacement of the probe, and $\varphi \left(\omega \right)$ is the phase shift between the applied force and the displacement. In creep measurements, a constant force is applied to the particle and the fluid compliance $J\left(t\right)$ is determined as:

(33) $$J\left(t\right)=6\pi a\frac{x\left(t\right)}{f}$$

where f is the magnitude of the applied force and $x\left(t\right)$ is the displacement of the particle over time. From here, $J^{*}\left(\omega \right)$ can be calculated [72]. Typical frequencies explored by MT experiments range from $10^{-2}$ to $10^{3}$ Hz, with shear moduli varying from $10^{-3}$ up to $10^{5}$ Pa.

**Figure 6:**
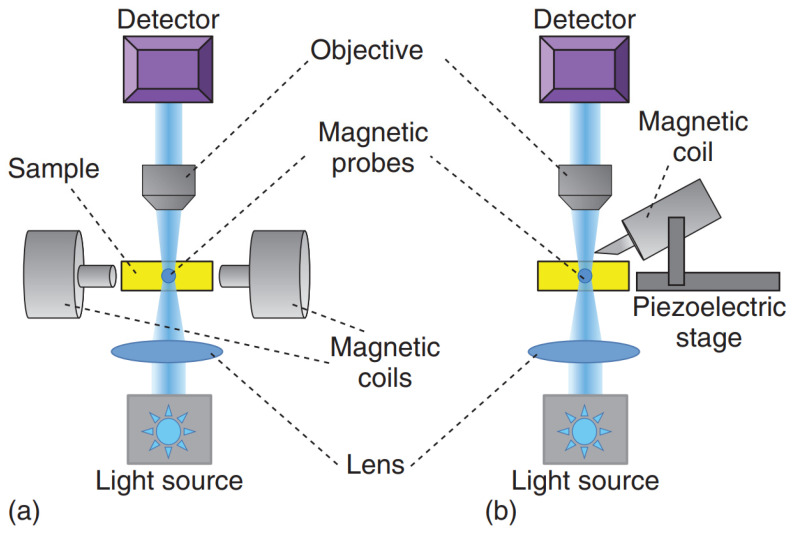
Schematic diagrams of two common set-ups used in magnetic tweezers microrheology experiments: (**a**) two aligned pole-pieces set-up; (**b**) single-coiled magnetic tweezers. Reprinted with permission from Ref. [48]. Copyright 2018 John Wiley & Sons.

**Figure 7:**
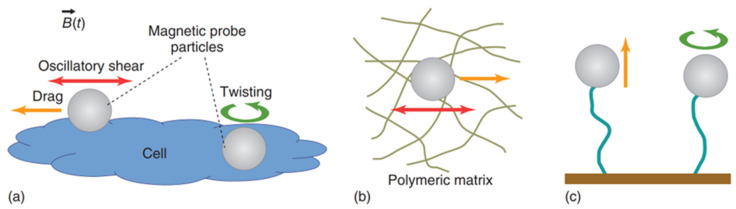
Schematic representations of different modus operandi of magnetic tweezers for microrheology studies: (**a**) single-cell mechanics via oscillatory, creep-compliance and twisting techniques; (**b**) oscillatory and creep-compliance experiments in viscoelastic media; (**c**) pulling and twisting methods applied to study the mechanical response of single molecules. Reprinted with permission from Ref. [48]. Copyright 2018 John Wiley & Sons.

It is important to mention that superparamagnetic beads are usually preferred over ferromagnetic ones due to their smaller hysteresis and the fact that ferromagnetic beads retain larger magnetic moments even when the external magnetic field is turned off. The calibration of MT can be done via the force–balance relation $\left|{\vec{F}}_{m}\left(t\right)\right|=\left|\vec{f_{v}}\left(t\right)\right|=6\pi \eta a\left|\vec{v}\left(t\right)\right|$ by considering experiments where probe particles with radius a are dragged in a fluid with known constant viscosity η (e.g., glycerol–water mixtures). Compared to optical tweezers, MT can provide larger forces (∼2000 pN), and they are less prone to heating the samples due to dissipation effects. However, MT do not allow the implementation of multiple independent traps. As schematically shown in Figure 6, both single-coiled and aligned two-pole-piece setups can be used to perform microrheology experiments. The first configuration is usually equipped with a piezoelectric stage and is mostly designed to perform creep-compliance experiments, whereas the second setup is commonly adopted to perform oscillatory experiments [5]. Figure 7 shows a schematic representation of different experimental methods for the most popular applications of magnetic tweezers, including alternative setups such as those used in magnetic twisting cytometry [73] and single-molecule experiments [74].

The seminal studies developed by Ziemann et al. [75] still play an important role in modern microrheology with MT. They demonstrated the viability of MT to perform both oscillatory and creep-compliance measurements and provided the theoretical framework to extract materials’ viscoelastic properties. In particular, they showed that different MT setups can yield quantitative measurements of entangled F-actin solutions, which displayed a power-law behaviour of the moduli $G^{\prime }∼G^{\prime \prime }\propto \omega^{1/2}$. A similar outcome has been achieved by microrheology with MT performed on many different cell lines.

Applications of oscillatory microrheology with MT also include one of the few in vivo microrheology experiments, where magnetic elongated probes were placed inside the intestinal bulb of a larval zebrafish. The results indicated that the larva’s mucin-rich intestinal liquid could be well described as a Newtonian fluid [76].

Alternative to oscillatory measurements with MT are microrheology studies based on creep-compliance experiments. Examples include early studies on the viscoelastic properties of adhering fibroblasts and J774 macrophages. These studies quantitatively showed that the mechanical response of cells is due to heterogeneous contributions from densely packed clusters and crosslinked filament networks separated by softer regions. In a more recent study, Yang et al. [77] used creep-compliance experiments to investigate the time- and force-dependent viscoelastic response of microtubule networks highly cross-linked by biotin–streptavidin bonds. Their results suggest that cross-linker binding/unbinding dynamics play a crucial role in the viscoelastic response of the network, displaying stiffening behaviour at short timescales while maintaining a soft response at long timescales due to the balance between bond breakage and formation.

For instance, Zakharov et al. [78] studied emulsions containing micron-sized droplets of hemoglobin to investigate sickle cell anemia through magnetically driven compression. Additionally, in a study related to thrombotic diseases, Whyte et al. [79] applied MT to show how fibrin polymerization is impaired by platelet-derived polyphosphate. They found that fibrin networks, essential to the mechanical properties and stability of blood clots, become softer and lose their structural function under these conditions.

Finally, it is worth mentioning an example of magnetic twisting cytometry (MTC) in the study of living cells. Hoffman et al. [80] used MT to investigate the frequency-dependent shear modulus of cultured mammalian cells (i.e., TC7 monkey kidney epithelial cells). They presented a comparative study that included other passive methods such as laser particle tracking (LPT) and two-point video particle tracking (VPT), clarifying the contributions of both cytoskeletal heterogeneities and ATP-dependent processes to the weak power law (n ∼ 0.17) of the viscoelastic moduli of mammalian cells.

### 3.3. Microrheology with Optical Tweezers

The advent of optical tweezers (OT) marked a pivotal moment in experimental science, thanks to Arthur Ashkin’s pioneering work in the 1970s [81]. OT exploit the interaction of highly focused laser beams with micron-sized dielectric particles, enabling three-dimensional trapping via gradients in light intensity. These exceptional transducers offer remarkable sensitivity, capable of resolving spatial displacements down to the nanometre scale, forces as small as piconewtons (pN), and temporal resolutions on the order of microseconds [82,83]. These capabilities have revolutionized microrheology, particularly in studying the viscoelastic properties of complex fluids and biological systems [48].

Accurate calibration of OT is essential for reliable microrheology measurements. The calibration process typically involves determining the trap stiffness κ using the principle of equipartition of energy:

(34) $$\frac{3}{2}k_{B}T=\frac{1}{2}\kappa \langle r^{2}\rangle$$

where $\langle r^{2}\rangle$ is the variance of the trapped particle’s position. This method ensures accurate force measurements and defines the operational range of the optical trap [82,84]. Alternatively, power spectral analysis of the particle’s motion can be employed to determine κ across different frequencies [85,86]. However, this latter procedure may be affected by the viscoelastic nature of the suspending media.

Optical tweezers are versatile tools that can be employed in both passive and active microrheology. In passive microrheology, the random motion of the trapped particle due to thermal fluctuations is confined by the harmonic potential of the optical trap. The dynamics of the particle in this regime are governed by the generalized Langevin equation:

(35) $$m\ddot{r}\left(t\right)=F_{R}\left(t\right)-\int_{-\infty }^{t}\zeta \left(t-t^{\prime }\right)\dot{r}\left(t^{\prime }\right)dt^{\prime }-\kappa r\left(t\right)$$

where m is the particle’s mass, $r\left(t\right)$ is the particle position, κ is the trap stiffness, and $F_{R}\left(t\right)$ is the usual Gaussian white noise term modelling stochastic thermal forces acting on the particle, the integral term, which incorporates a generalized time-dependent memory function $\zeta \left(t\right)$, represents viscous damping by the fluid.

**Figure 8:**
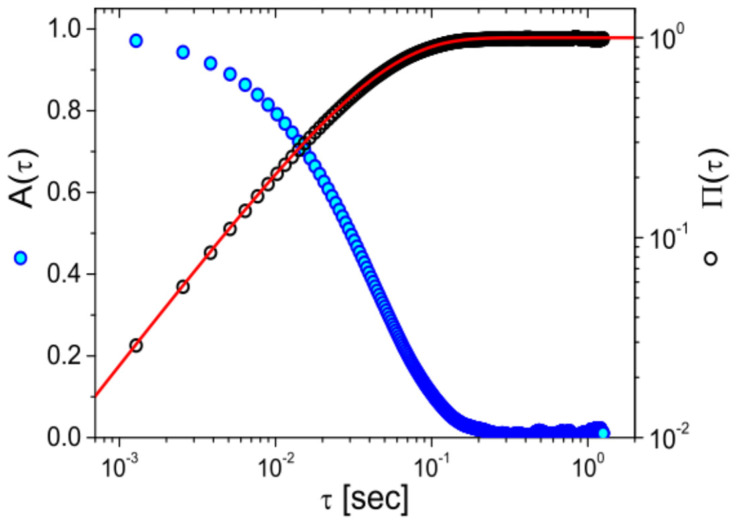
The normalized position autocorrelation function $A\left(\tau \right)$ and the normalized mean square displacement $\Pi \left(\tau \right)$ versus lag-time τ for an optically trapped particle suspended in water at room temperature. The continuous line is the theoretical prediction: $\Pi \left(\tau \right)=1-A\left(\tau \right)=1-e^{-\frac{\tau }{t^{*}}}$, with $t^{*}=\frac{6\pi a\eta }{\kappa }$.

Building on the assumptions established by Mason and Weitz [3] for freely diffusing particles, the frequency-dependent complex shear modulus ($G^{*}\left(\omega \right)$) of a material can be determined via either the normalized mean squared displacement (NMSD), $\Pi \left(\tau \right)=\frac{\left\langle \Delta r^{2}\left(\tau \right)\right\rangle }{2\left\langle r^{2}\right\rangle }$ [87], or the normalized position autocorrelation function (NPAF), $A\left(\tau \right)=\frac{\left\langle r\left(t\right)\cdot r\left(t+\tau \right)\right\rangle }{\left\langle r^{2}\right\rangle }$ [83] shown in Figure 8.

The relationship is given by:

(36) $$G^{*}\left(\omega \right)\frac{6\pi a}{\kappa }=\left(\frac{1}{i\omega \hat{\Pi }\left(\omega \right)}-1\right)\equiv {\left(\frac{1}{i\omega \hat{A}\left(\omega \right)}-1\right)}^{-1}\equiv \frac{\hat{A}\left(\omega \right)}{\hat{\Pi }\left(\omega \right)}$$

where $\hat{\Pi }\left(\omega \right)$ and $\hat{A}\left(\omega \right)$ are the Fourier transforms of $\Pi \left(\tau \right)$ and $A\left(\tau \right)$, respectively. The inertial term ($m\omega^{2}$) from the original works is omitted here because for micron-sized particles (of radius a) it becomes relevant only at frequencies of the order of MHz range [7]. Additionally, for sufficiently long measurements, it can be shown that $\Pi \left(\tau \right)=1-A\left(\tau \right)$; which holds also in the frequency domain, $i\omega \hat{\Pi }\left(\omega \right)=1-i\omega \hat{A}\left(\omega \right)$.

**Figure 9:**
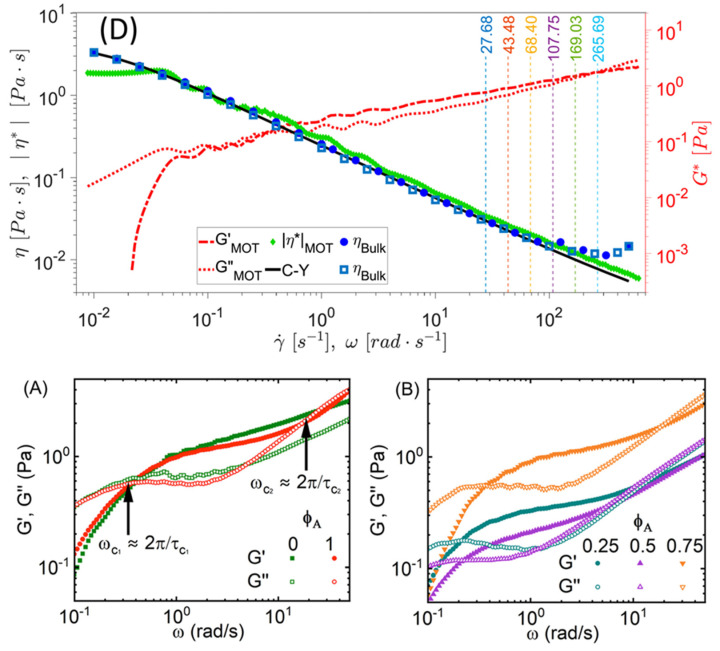
(**Top**, originally labelled as ‘D’ in the source publication.) Comparison between the shear viscosity $\eta \left(\dot{\gamma }\right)$ and the complex viscosity $\eta^{*}\left(\omega \right)$—derived from the frequency-dependent viscoelastic moduli—of a polyacrylamide solution. The agreement spans over five decades of shear rate and frequency. Reprinted with permission from Ref. [88]. Copyright 2023 AIP. (**Bottom**) (**A**,**B**) Viscoelastic response of actin–vimentin composite networks, highlighting the interplay between actin stiffness and vimentin extensibility. Reprinted with permission from Ref. [89]. Copyright 2024 Royal Society of Chemistry.

The application of Equation (36) has proven invaluable for broadband microrheology measurements of complex fluids, as recently demonstrated by Smith et al. [88] and Pinchiaroli et al. [89]. Smith et al. [88] employed this approach to analyze the viscoelastic properties of polyacrylamide solutions, revealing two characteristic crossover frequencies in the fluid’s moduli, indicative of distinct relaxation processes (Smith et al. [88], Figure 9). Similarly, Pinchiaroli et al. utilized optical tweezers microrheology to investigate actin-vimentin composites, capturing the interplay between actin’s stiffness and vimentin’s extensibility, which resulted in emergent mechanical behaviours in both linear and nonlinear regimes (Pinchiaroli et al. [89], Figure 9). These studies underscore the versatility of microrheological techniques for elucidating the intricate mechanical characteristics of complex systems across diverse spatial and temporal scales.

**Figure 10:**
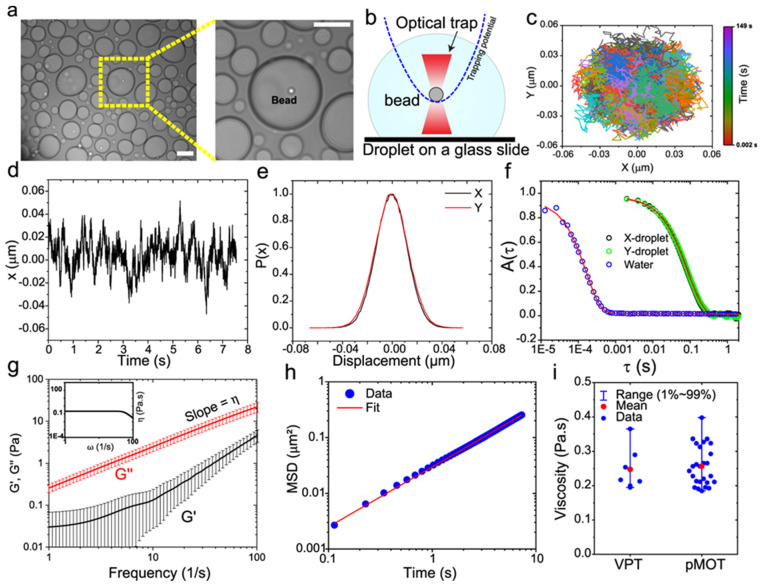
Determination of frequency-dependent viscoelastic moduli of peptide-RNA condensates using passive microrheology with optical tweezers (pMOT). (**a**) A bright-field image showing a polystyrene bead (1 µm) trapped within a [KGKGG]5-rU40 condensate using an optical trap. Scale bar = 10 µm. (**b**) A conceptual scheme of the pMOT experiment. The bead is optically trapped within a biomolecular condensate sitting on a microscope glass surface. (**c**) A representative 2D trajectory of the bead shown in (**a**) within the optical trap inside a [KGKGG]5-rU40 condensate. (**d**) The trajectory of the trapped bead in the X-direction. (**e**) Normalized distribution of displacements along the X- and Y-directions for the trajectory in (**c**,**d**). (**f**) The normalized position autocorrelation function [NPAF, A(t)] as calculated from the trajectory in (**c**) for a bead that is optically trapped inside [KGKGG]5-rU40 condensate (green and black) and inside water (blue) as a reference. Solid lines are multi-exponential fits (see Supplementary Note 1 of the original manuscript). (**g**) The average viscoelastic moduli as obtained from normalized position autocorrelation function using Equation (1) (of the original manuscript) for [KGKGG]5-rU40 condensates. $G^{\prime }$ and $G^{\prime \prime }$ represent the elastic and viscous modulus, respectively. Solid lines are averages of the moduli of 10–20 condensates. Error bars represent the standard deviation as calculated from the moduli of 10–20 condensates. Inset: frequency-dependent condensate viscosity as determined from the viscous modulus using the relation $\eta \left(\omega \right)=\frac{G^{\prime \prime }\left(\omega \right)}{\omega }$. (**h**) The ensemble-averaged mean square displacement (MSD) of 200 nm polystyrene beads within [KGKGG]5-rU40 condensates using video particle tracking (VPT) microrheology in absence of optical traps (see Methods section for further details). (**i**) Comparison between the zero-shear viscosity as determined by pMOT- (n = 26 measurements over 3 independent samples) and VPT-derived (n = 7 measurements over 3 independent samples) viscosity. Error bars represent the range of the data. Reprinted with permission from Ref. [90]. Copyright 2021 Springer Nature.

Building on this foundation, recent studies have leveraged optical tweezers to probe the viscoelastic properties of biomolecular condensates, further expanding the scope of microrheological applications. Alshareedah et al. [90] employed this method to investigate peptide-RNA condensates seen in Figure 10, demonstrating how sequence-specific interactions between “sticker” and “spacer” residues govern transitions between elastic and viscous states. By tuning these molecular features, they engineered condensates with programmable mechanical properties. Meanwhile, Alshareedah et al. [91] explored protein condensates derived from prion-like domains, revealing age-dependent transitions from Maxwell fluid-like behaviours to semi-crystalline elastic solids. This progression, driven by aromatic residue interactions, provided critical insights into condensate stability and functionality. Together, these works highlight the power of optical tweezers microrheology in uncovering the molecular determinants of complex viscoelastic behaviours, enabling both fundamental discoveries and the design of materials with tailored mechanical responses.

Active microrheology with optical tweezers (MOT) typically involves oscillatory measurements, akin to those used in conventional bulk rheology. However, for MOT applied to complex fluids, the expression for $G^{*}\left(\omega \right)$ must be derived by solving a generalized Langevin equation. This equation extends Equation (35) by incorporating an additional term to account for the motion of a nonstationary trap:

(37) $$m\ddot{r}\left(t\right)=F_{R}\left(t\right)-\int_{-\infty }^{t}\zeta \left(t-t^{\prime }\right)\dot{r}\left(t^{\prime }\right)dt^{\prime }-\kappa \left(r_{c}\left(t\right)-r\left(t\right)\right)$$

where all terms are as defined in Equation (35), with the addition of $r_{c}\left(t\right)$, the position vector describing the driven motion of the optical trap centre. The solution to this equation provides $G^{*}\left(\omega \right)$ in terms of the particle position $r\left(t\right)$, expressed as follows [83]:

(38) $$G^{*}\left(\omega \right)\frac{6\pi a}{\kappa }=\left(\frac{\langle {\hat{r}}_{c}\left(\omega \right)\rangle }{\langle \hat{r}\left(\omega \right)\rangle }-1+\frac{m\omega^{2}}{\kappa }\right)$$

where ${\hat{r}}_{c}\left(\omega \right)$ and $\hat{r}\left(\omega \right)$ are the Fourier transforms of $r_{c}\left(t\right)$ and $r\left(t\right)$, respectively, and $\langle \cdots \rangle$ denotes averaging over multiple independent measurements. Importantly, this equation is generally valid regardless of the temporal form of $r_{c}\left(t\right)$.

**Figure 11:**
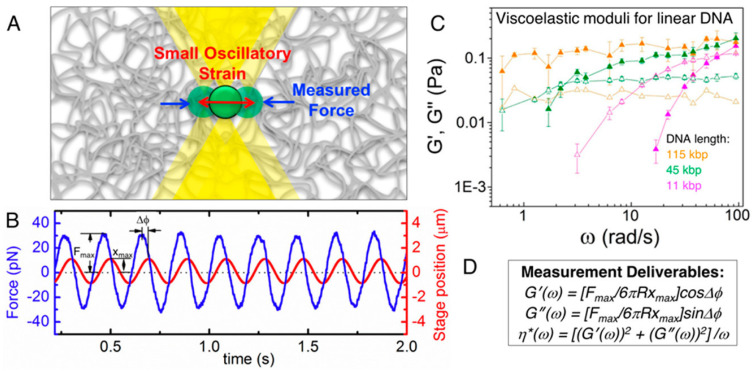
Linear oscillatory microrheology. (**A**) An optically trapped microsphere is sinusoidally displaced through the sample while the force exerted on the bead is measured. (**B**) Sample data showing the position of the stage (which moves the trap relative to the sample) and the measured force during oscillation. The stage amplitude x_max_, force amplitude F_max_, and phase shift Δϕ between the two curves for each frequency ω are measured to compute the linear viscoelastic moduli. (**C**) G′(ω) (closed symbols) and G″(ω) (open symbols) measured using this method for 1 mg/mL linear DNA of varying lengths (listed in legend). Reprinted with permission from Ref. [93]. Copyright 2014 ACS. (**D**) Equations relating measured quantities to viscoelastic moduli for a microsphere of a given radius R. Reprinted with permission from Ref. [92]. Copyright 2018 American Chemical Society.

Robertson-Anderson’s extensive work, summarized in her comprehensive review and shown in Figure 11, highlights the transformative potential of active MOT for studying the viscoelastic properties of complex fluids. Through controlled oscillatory and displacement-driven measurements, MOT provides a powerful tool for quantifying frequency-dependent storage and loss moduli ($G^{\prime }\left(\omega \right)$ and $G^{\prime \prime }\left(\omega \right)$) by tracking the motion of a trapped bead under sinusoidal displacement. This technique has been instrumental in uncovering the microscale viscoelastic responses of macromolecular networks such as DNA and actin, enabling insights into both linear and nonlinear rheological behaviour. Furthermore, MOT’s precision allows the study of spatially resolved dynamics and stress propagation, bridging the gap between molecular interactions and bulk material properties [92,93].

### 3.4. Microrheology with Dynamic Light Scattering

**Figure 12:**
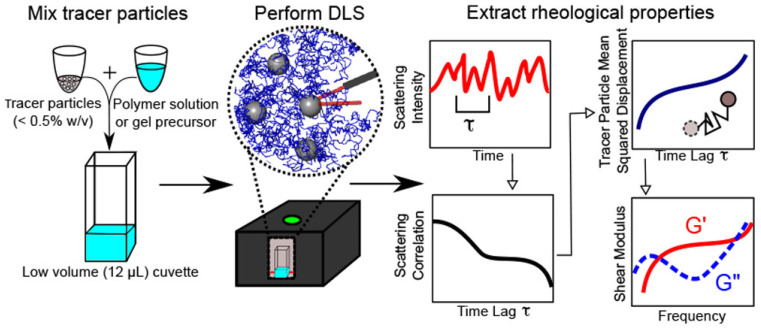
DLS microrheology workflow. The polymer solution or gel precursor is mixed with a dilute concentration of tracer particles (<0.5% *w*/*v*). DLS is performed in a backscattering configuration using a commercial benchtop instrument. Brownian motion of the tracer particles produces fluctuations in scattering intensity that give rise to a characteristic scattering intensity autocorrelation. The autocorrelation is analyzed by our custom software to extract the mean-squared displacement of particles, which is used to determine the frequency-dependent linear viscoelastic shear modulus G*(ω). Reprinted with permission from Ref. [29]. Copyright 2017 American Chemical Society.

Microrheology with Dynamic Light Scattering (DLS) is a powerful technique that plays a pivotal role in probing the dynamics of particles in suspension and extracting the viscoelastic properties of complex fluids. Also known as photon correlation spectroscopy (PCS) or quasi-elastic light scattering (QELS), DLS measures the distribution of relaxation times in the scattered field autocorrelation function $g_{1}\left(\tau \right)$ from colloidal and macromolecular solutions [94]. A typical workflow for obtaining the rheological properties from polymer or gel precursors through DLS is depicted in Figure 12.

In a typical DLS experiment, the scattered intensity $I\left(t\right)$ is collected with a detector, and the intensity autocorrelation function $g_{2}\left(\tau \right)$ is evaluated using a correlator. The intensity autocorrelation function is mathematically defined as:

(39) $$g_{2}\left(\tau \right)=\frac{\left\langle I\left(t\right)I\left(t+\tau \right)\right\rangle }{{\left\langle I\left(t\right)\right\rangle }^{2}}$$

where $I\left(\tau \right)$ represents the light intensity at time τ, and $\left\langle \cdots \right\rangle$ denotes a time average. The electric field autocorrelation function $g_{1}\left(\tau \right)$ is derived from the Siegert relation:

(40) $$g_{2}\left(\tau \right)=1+\beta {\left|g_{1}\left(\tau \right)\right|}^{2}$$

where β is a constant that varies with the experimental setup and must be determined empirically. This relation is fundamental to understanding the dynamics of colloidal particles undergoing Brownian motion. In the single scattering limit, $g_{1}\left(\tau \right)$ for an ensemble of such particles can be expressed as:

(41) $$g_{1}\left(\tau \right)=\exp\left(-\frac{1}{6}q^{2}\left\langle \Delta r^{2}\left(\tau \right)\right\rangle \right)$$

Here, $q=\frac{4\pi n}{\lambda }\sin\left(\theta /2\right)$ is the scattering vector, where λ is the wavelength of light, n is the refractive index of the medium, and θ is the scattering angle [37]. The mean square displacement (MSD) $\left\langle \Delta r^{2}\left(\tau \right)\right\rangle$ of the particles can then be related to the autocorrelation function $g_{1}\left(\tau \right)$, providing insights into the viscoelastic properties of the material under study.

DLS is particularly useful for evaluating the linear viscoelastic (LVE) properties of materials across a wide frequency range, from approximately 0.1 Hz to 1 MHz [95]. It captures the 3D motion of probe particles, offering comprehensive insights compared to other microrheology techniques like particle-tracking microrheology [83]. However, DLS-microrheology is most effective in situations where single scattering events dominate the autocorrelation function $g_{2}\left(\tau \right)$. This requirement often necessitates a low concentration of probe particles to prevent multiple scattering and a higher intensity of scattered light from the probes than from the sample’s other components, such as protein filaments [29].

In cases involving gel-like materials, which still operate within the single scattering regime, static contributions to the scattering intensity autocorrelation function can become significant. This scenario may require replacing $g_{1}\left(\tau \right)$ with $g_{1}^{gel}\left(\tau \right)$ in the MSD equation to account for these contributions [96]. Despite these complexities, DLS-microrheology has been successfully employed in various studies. For example, it was used to investigate water-based hydroxyethyl cellulose (HEC) solutions, where results showed excellent agreement with other rheological techniques [95].

**Figure 13:**
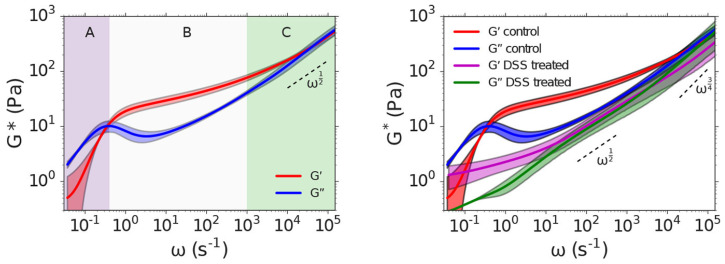
DLS microrheology captures the entangled dynamics of intestinal mucus of healthy and colitic mice. (**Left**): Dependence of the shear modulus G* on angular frequency ω of intestinal mucus isolated from healthy mice. The shear modulus exhibits three regimes, A, B, and C, which we identify as corresponding to reptation of polymers within an entangled network, elastic behaviour due to entanglement constraints, and Rouse-like flexible chain dynamics at length scales below the entanglement confinement length, respectively. Solid curves represent the mean among 3 independent biological reproductions. Shading represents 90% confidence intervals of the mean generated by bootstrap resampling spectra from independent biological reproductions. The dashed line represents the high-frequency scaling behaviour of a Rouse polymer G* ∼ ω^1/2^, which is provided to guide the eye. (**Right**): Comparison of the frequency-dependent shear modulus G* of intestinal mucus isolated from healthy (control) mice and mice treated with dextran sulphate sodium (DSS) to induce colitis. The high frequency scaling behaviours of a Rouse polymer and a WLC (G* ∼ ω^1/2^ and G* ∼ ω^3/4^, respectively) are indicated with dashed lines for reference. Reprinted with permission from Ref. [29]. Copyright 2017 American Chemical Society.

Furthermore, Krajina et al. [29] demonstrated the utility of DLS in studying biological systems, including polyacrylamide gels and semiflexible polymers like DNA. Their work also highlighted differences in the viscoelastic properties of intestinal mucus from healthy and colitic mice as seen in Figure 13, showing that the mucus from colitic mice was significantly softer. These findings underscore DLS-microrheology as a versatile and powerful tool for studying the microrheological properties of both synthetic and biological materials.

### 3.5. Microrheology with Diffusing Wave Spectroscopy

Diffusing Wave Spectroscopy (DWS) is an advanced light scattering technique developed to analyze the structure and dynamics of turbid materials, where traditional single-scattering approaches are ineffective. Originating from the work of Weitz and Pine in the late 1980s, DWS extends the principles of photon correlation spectroscopy into the multiple scattering regime. This makes it possible to study a wide range of complex systems, including foams, emulsions, and gels, where single scattering assumptions break down [97,98].

DWS is based on measuring the electric field autocorrelation function, $g_{1}\left(\tau \right)$, which describes the time-dependent behaviour of light scattered through a sample. In systems where multiple scattering dominates, the propagation of light is described by a diffusive model. The scattered light undergoes numerous scattering events, effectively following a random walk within the sample before being detected. The autocorrelation function in DWS is given by:

(42) $$g_{1}\left(\tau \right)=\int_{0}^{+\infty }P\left(s\right)\exp\left[-\frac{sk_{0}^{2}\left\langle \Delta r^{2}\left(\tau \right)\right\rangle }{3l^{*}}\right]ds$$

where $k_{0}=\frac{2\pi n}{\lambda }$ is the wave vector of the incident light (with n as the refractive index and λ as the wavelength), $l^{*}$ is the transport mean free path of the light, $\left\langle \Delta r^{2}\left(\tau \right)\right\rangle$ is the mean squared displacement (MSD) of the probe particles, and $P\left(s\right)$ represents the probability distribution of path lengths that the photons travel.

For the light propagation to be diffusive, the condition $l^{*}\ll L$ (where *L* is the sample thickness) must be satisfied. The mean free path $l^{*}$ can be determined using a reference fluid with known viscosity by measuring the transmission *T* of light through the sample:

(43) $$T=\frac{\frac{{5l}^{*}}{3L}}{1+\frac{4l^{*}}{3L}}≅\frac{{5l}^{*}}{3L}$$

The choice of geometry in DWS experiments, either transmission or backscattering, significantly affects the path lengths probed. In the transmission geometry, the setup primarily captures long path lengths, enabling the study of fast dynamics. The electric field autocorrelation function for a point source in transmission geometry, for which the instantaneous source of diffusing intensity has been taken at point $\left(x_{0},\;y_{0},z_{0}\right)$ and the detected light is collected from the multiply scattered light emerging from the sample at $\left(x,\;y,z\right)=\left(0,\;0,L\right)$, can be expressed as:

(44) $$g_{1}\left(\tau \right)=\;C\int_{Q}^{\infty }J_{0}\left.\left(\frac{R}{L}\sqrt{\left\{\xi^{2}-\;Q^{2}\right\}}\right.\right)G\left(\xi ,\;\epsilon ,\;\zeta \right)\xi e^{-\left(1\;-\;\zeta \right)\xi }d\xi$$

where C is a normalization constant chosen so that $g_{1}\left(0\right)=1$, $J_{0}$ is flux of photons arriving at the point $\left(x_{0},y_{0},z_{0}\right)$, $Q=\left(\frac{L}{l^{*}}\right)\sqrt{\frac{6\tau }{\tau_{0}}}$, $R^{2}={\left(x-x_{0}\right)}^{2}+{\left(y-y_{0}\right)}^{2}$, $\epsilon =2l^{*}/\left(3L\right)$, $\zeta =z_{0}/L$, $\tau_{0}={\left(k_{0}D\right)}^{-1}$, D is the diffusion coefficient, and $G\left(\xi ,\epsilon ,\zeta \right)$ is a function that accounts for the sample’s thickness L and the mean free path $l^{*}$ [99].

To facilitate ensemble averaging in non-ergodic systems, a two-cell reference setup is often used. This setup involves placing a reference cell before the sample to average out correlation functions. The autocorrelation function for the combined system is described by:

(45) $$g_{1}^{\left(2\right)}\left(L_{1},L_{2},\tau \right)\approx g_{1}^{\left(1\right)}\left(L_{1},\tau \right)\cdot g_{1}^{\left(1\right)}\left(L_{2},\tau \right)$$

**Figure 14:**
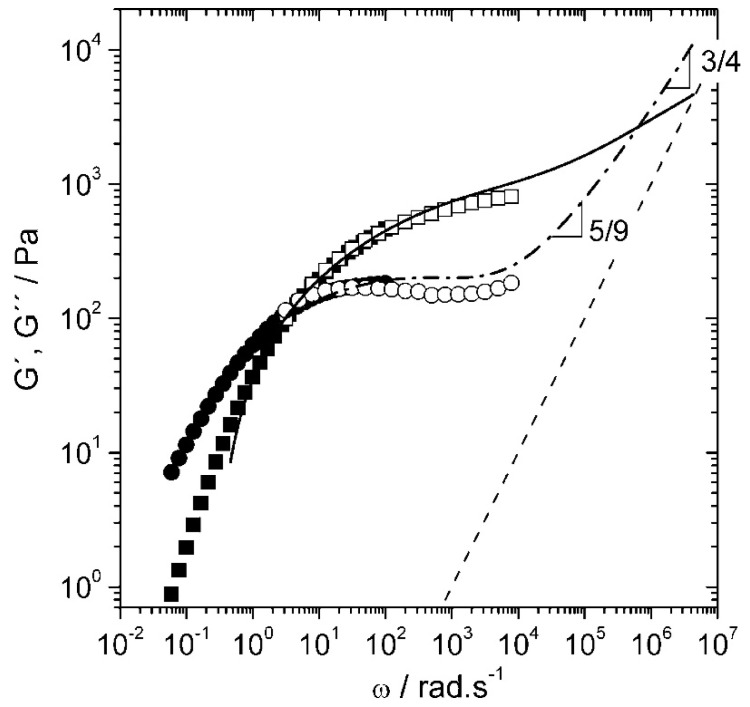
Dynamic shear moduli G′ and G″ of a 30 g/L NaHA solution in presence of 0.1 M NaCl obtained from DWS after inertial correction (G′ solid line, G″ dash-dotted line), oscillatory squeeze flow (G′ open squares, G″ open circles), and rotational rheometry (G′ closed squares, G″ closed circles) at T = 20 °C. The modulus of water G″ = ωη_s_ is included for reference (dashed line). Reprinted with permission from Ref. [100]. Copyright 2013 American Chemical Society.

DWS is highly effective for probing a broad range of frequencies, from 0.1 Hz  to 1 MHz, enabling the study of complex viscoelastic behaviour in various materials. Notable applications include measuring the high-frequency linear viscoelasticity of biopolymer networks, studying the dynamics of sheared colloidal suspensions, and analyzing the mechanical properties of phase-separated polymer systems [98]. For instance, DWS, along with high-frequency mechanical rheology, has been used to characterize the viscoelastic properties of hyaluronan solutions over a broad range of frequencies as seen in Figure 14. The persistence length of hyaluronan solutions was then obtained from the semi-dilute entangled regime [100].

Recently, good agreement between bulk rheology and DWS measurements were demonstrated by Li et al. [38] without the incorporation of tracer particles, or where particles within the suspension themselves were responsible for light scattering. They found that using a two-point interpretation of the GSER, which considers the correlated motion between particles, was applicable to samples where the average scattering vector corresponding to photon propagation is larger than the diameter of the scattering centres.

### 3.6. Atomic Force Microscopy

**Figure 15:**
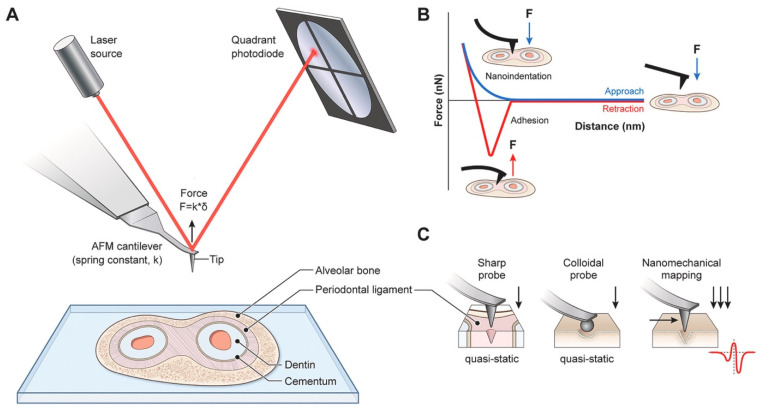
Fundamental principles of Atomic Force Microscopy (AFM)-based tissue mechanobiology. (**A**) Schematic describing the general AFM principles. An infrared laser is focused onto a soft microcantilever and its reflection is detected by a four-quadrant photodetector. When the microcantilever is in physical contact with the tissue specimen, the cantilever deflection is detected by the movement of the laser spot in the photodetector. (**B**) Diagram illustrating a typical force against Z-distance curve performed on a tissue specimen. The blue curve is the approach curve and indicates the resistance of the tissue to deform when the indenter is pushing against the tissue. The red curve is the retraction curve and indicates viscous relaxation and the adhesion of the tissue. (**C**) The illustrations indicated typical nanomechanical AFM-based mapping modalities used to measure the mechanical properties of tissues at high spatiotemporal resolution. (For interpretation of the references to colour in this figure legend, the reader is referred to the Web version of this article). Reprinted with permission from Ref. [101]. Copyright 2023 Elsevier.

Atomic Force Microscopy (AFM) is a highly versatile technique designed for imaging, measuring, and manipulating materials at nanometric scales. Central to its operation is a sharp tip affixed to a cantilever, which interacts with the sample surface through forces such as van der Waals, electrostatic, and hydrodynamic forces. A piezoelectric actuator precisely controls the cantilever’s movement, enabling force application while maintaining surface integrity. The quadrant photodiode detector measures cantilever deflection to nanometre precision, providing insights into surface topology and mechanical properties. A summary of the working principles of AFM is found in Figure 15 [101].

AFM has been extensively utilized to measure the frequency-independent Young’s modulus of materials by analyzing force–indentation (F-δ) curves. These curves are model-dependent, requiring theoretical frameworks such as the Hertz-Sneddon or Johnson-Kendall-Roberts models for data interpretation. For purely elastic surfaces, when using a spherical indenter, the force–indentation relationship is expressed as follows:

(46) $$F\;=\frac{3ER^{1/2}}{4\left(1\;-\;\nu^{2}\right)}\delta^{3/2}$$

where E is Young’s modulus, R the indenter radius, ν Poisson’s ratio, and δ the indentation depth.

However, it is important to recognize that most real materials exhibit viscoelastic behaviour, possessing both viscous and elastic components. Consequently, limiting the analysis to the evaluation of the frequency-independent Young’s modulus alone can result in a significant loss of critical information. Such information has proven to be essential for accurately describing the response of biological systems, such as cells, to the viscoelastic properties of their surrounding environment [102,103].

To address this gap in knowledge, Tassieri et al. [104] developed an analytical framework grounded in the analysis of stress-relaxation data. This framework is based on the same principles underlying the innovative rheological tool, “i-Rheo” [105], which enables the evaluation of a material’s linear viscoelastic properties over an exceptionally broad range of experimentally accessible frequencies, derived from a simple time-dependent step-strain measurement. In the context of AFM, experiments provide time-resolved measurements of force $F\left(t\right)$ and indentation $\delta \left(t\right)$. The constitutive equation for linear viscoelasticity, which forms the theoretical basis of this methodology, operates on the principle that the effects of sequential changes in indentation are additive [45,106]. Mathematically, this relationship is expressed as:

(47) $$F\left(t\right)=\int_{-\infty }^{t}G\left(t-t^{\prime }\right)\dot{\Lambda }\left(\delta \left(t^{\prime }\right)\right)dt^{\prime }$$

where $G\left(t\right)$ is the material’s shear relaxation modulus and $\dot{\Lambda }\left(\delta \left(t\right)\right)$ is the time derivative of $\Lambda \left(\delta \left(t\right)\right)=\frac{8\sqrt{R}}{3\left(1\;-\;\nu \right)}\delta^{\frac{3}{2}}\left(t\right)$. This formulation allows the extraction of viscoelastic properties directly from the time-domain data, ensuring a robust and comprehensive characterization of the material’s response. Equation (47) represents a convolution integral between the two time-dependent functions, $G\left(t\right)$ and $\Lambda \left(t\right)$. Applying the Fourier transform to this equation converts the convolution into a product of the Fourier transforms of these functions. Consequently, in the frequency domain, Equation (47) can be expressed as:

(48) $$G^{*}\left(\omega \right)=\frac{\hat{F}\left(\omega \right)}{\hat{\Lambda }\left(\omega \right)}$$

**Figure 16:**
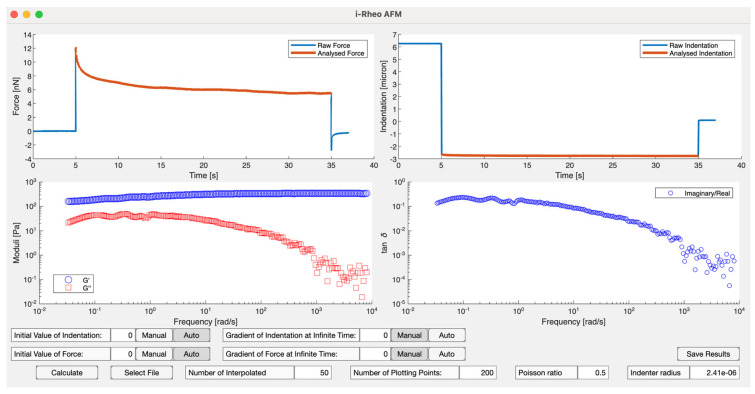
The frontend of the open-access graphical user interface (GUI) developed by Haidar and Tassieri [107]. This code processes step-indentation measurements and provides the frequency-dependent viscoelastic moduli of the material. The stress-relaxation nanoindentation procedure involves approaching the sample surface, performing the indentation to a predefined depth, and holding it for a set duration while recording force deflection (red line). Subsequently, the cantilever is retracted from the surface.

This formulation provides a comprehensive description of the material’s viscoelastic properties in the frequency domain. To minimize computational burden, Haidar and Tassieri [107] have developed an open-access MATLAB-based code designed to evaluate the viscoelastic properties of materials through the analysis of step-indentation measurements performed using atomic force microscopy, as seen in Figure 16.

### 3.7. Synergizing Passive and Active Techniques: Advancing Broadband Microrheology

**Figure 17:**
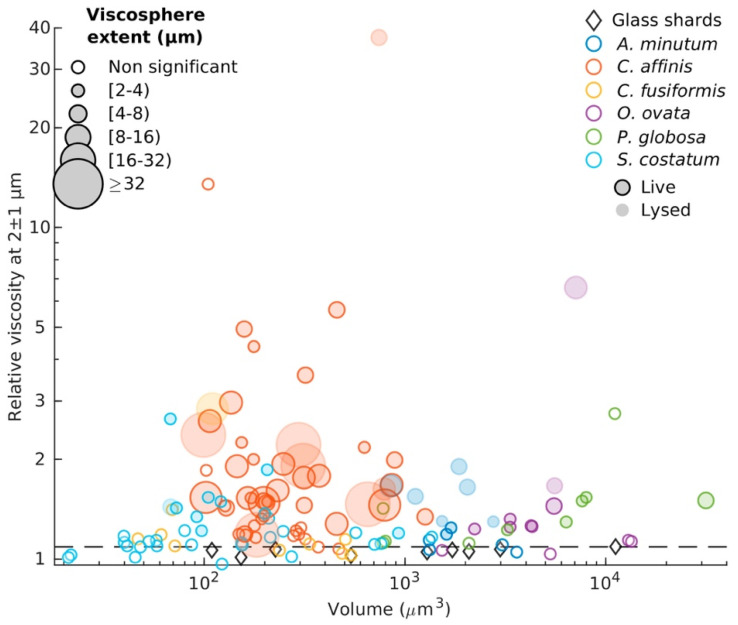
Average relative viscosities between 1 and 3 µm away from cells in relation to cells volume. The diameter of each symbol is proportional to the extent of the viscous phycosphere. Horizontal dashed line represents predictions from Faxén’s law for motion perpendicular to a solid boundary (Equation (2)). Reprinted with permission from Ref. [108]. Copyright 2021 National Academy of Sciences.

The evolution of particle tracking microrheology over recent years has been marked by significant advancements in both methodology and application, enabling deeper exploration of biological systems. Starting with its application in marine environments, a 2021 study revealed the existence of microscale viscosity gradients in planktonic systems, highlighting how microbial activity creates spatial heterogeneities known as “viscospheres” shown in Figure 17. These gradients, formed by extracellular polymeric substances (EPS) surrounding phytoplankton cells, were shown to influence nutrient uptake, chemotaxis, and microbial interactions, thereby impacting carbon and nutrient cycles on a global scale. This innovative approach underscored the critical role of microrheology in understanding ecological dynamics at microscale levels [108].

**Figure 18:**
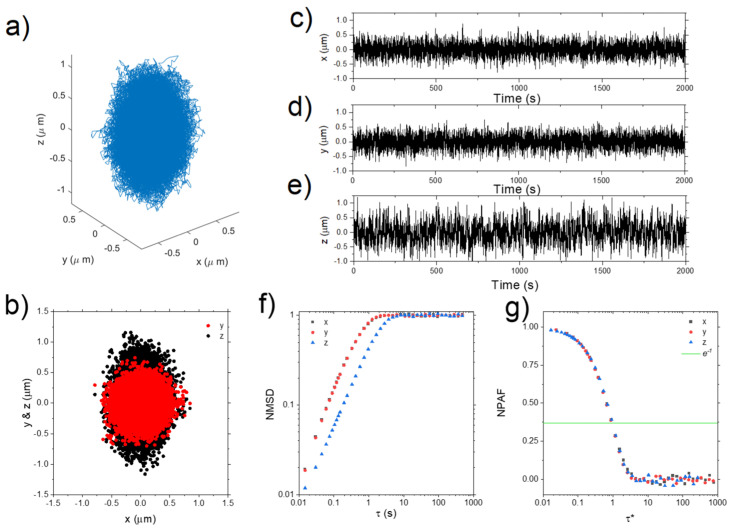
(**Left**) A single frame showing a bead of ~ 4 µm radius trapped in gel and imaged at nine depths simultaneously. The top-left corner of each image reports the relative distance between that specific image plane and the plane in the centre (0), in multiples of the plane spacing Δz = 0.88 µm. The scale bar, shown in the central plane (0) is 10 µm. Contrast and brightness have been adjusted for clarity (MATLAB 2019b; MathWorks, Natick, MA, USA, https://uk.mathworks.com/products/matlab.html). (**Right**) (**a**) 3D scatter plot of the trajectory of a ~ 7 µm diameter bead confined in space by an optical trap (MATLAB 2019b; MathWorks, Natick, MA, USA, https://uk.mathworks.com/products/matlab.html). (**b**) Projections of the trajectory on the x–y and x–z planes. The bead trajectory is drawn from the image analysis of ~ 100,000 frames. (**c**–**e**) show the x, y, and z position, respectively, of the bead with time over the length of the experiment. (**f**) The bead NMSD versus lag-time τ evaluated for each dimension. (**g**) The particle NPAF for each dimension plotted against a dimensionless lag-time τ*, derived from the scatter plots shown in (**a**). The solid line is at NPAF = e^−1^. Reprinted with permission from Ref. [109]. Copyright 2021 Springer Nature.

In the same year, the development of Optical Tweezers with Integrated Multiplane Microscopy (OpTIMuM) represented a major leap forward in 3D microrheology. By combining optical tweezers with multiplane microscopy, OpTIMuM enabled precise tracking of particle movements in three dimensions with nanometre accuracy, as seen in Figure 18. This tool not only enhanced the capability to measure the viscoelastic properties of fluids in complex biological environments but also eliminated the need for extensive calibrations. Its simplicity and versatility made it invaluable for studying the mechanical properties of anisotropic materials and complex interfaces, opening new possibilities for biomedical research [109].

**Figure 19:**
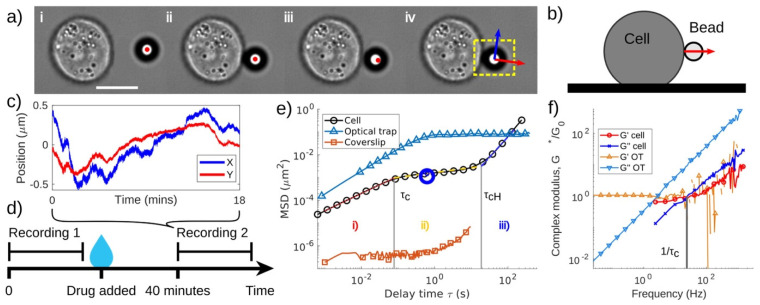
Data collection and interpretation. (**a**) Experimental procedure as described in text. Red and blue arrows denote radial and tangential directions, respectively. Yellow box shows region of interest. Scale bar in i is 10 µm. (**b**) Schematic side view of bead attached to cell. (**c**) Position–time trace from 1 measurement; the 18 min trace consists of 2,000,000 observations. (**d**) Experimental paradigm for change over time: two video measurements are taken 40 min apart with a drug being added after the first measurement, note that other measurements may have been made in this window, but only two are compared. (**e**) log–log plot with three example MSD curves: typical data from bead attached to cell, optically trapped bead, and bead attached to coverslip (to demonstrate noise floor). Three regions are highlighted for the cell MSD: (i) viscoelastic response at short time, (ii) soft glassy plateau at intermediate time with power-law exponent minima indicated by blue circle, and (iii) superdiffusion at long times. (**f**) Normalized complex modulus of a cell (Equation (3)), and of optical tweezers, calculated from the Fourier transform of the normalized MSD. Characteristic time scales found empirically are labelled one) and (**f**). (For interpretation of the references to colour in this figure legend, the reader is referred to the web version of this article.) Reprinted with permission from Ref. [63]. Copyright 2023 Elsevier.

Building on this foundation, a 2023 study introduced a novel passive microrheology technique that used living cells as biological analogues of optical tweezers. This method, summarized in Figure 19 involved chemically binding beads to the surfaces of live cells to monitor their dynamic mechanical properties during processes such as cytoskeletal rearrangement and adhesion maturation. The study provided groundbreaking insights into cell stiffening, softening, and mechanotransduction, all of which are critical for understanding disease progression and developing therapeutic strategies. This minimally invasive approach allowed for long-term monitoring of cellular biomechanics, significantly advancing the study of live cell mechanics [63].

**Figure 20:**
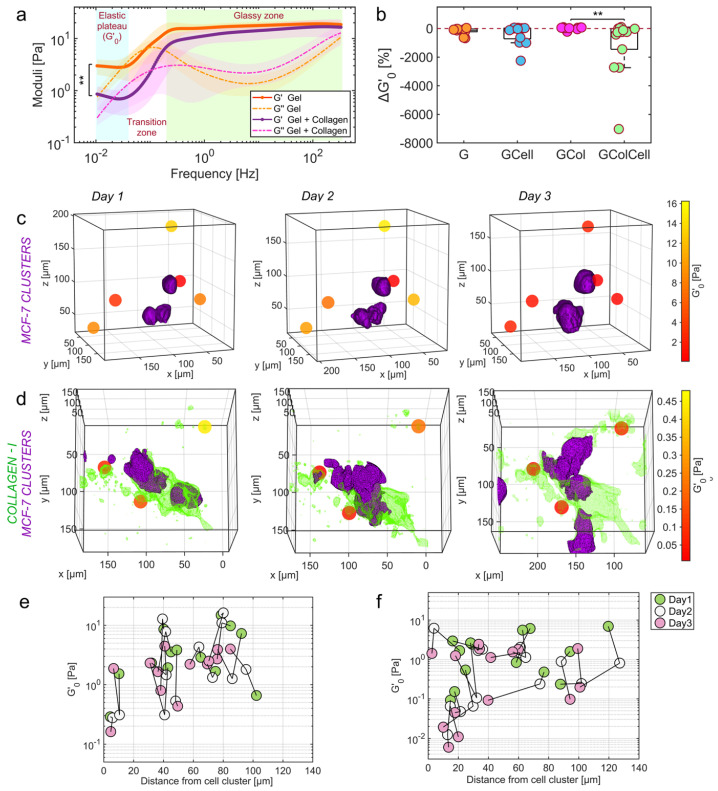
Local stiffness measured in live 3D cell cultures with different compositions. (**a**) Complex moduli of plain gel in orange and gel supplemented with collagen in purple at day 1 (n = 11 for each condition averaged with 95% confidence intervals shown as shaded regions). (**b**) Proportional change in the height of the low frequency elastic plateau $G_{0}^{\prime }$ (Equation (4) in Methods) at individual bead probes over three days of observation in plain gel (G) n = 11, gel with collagen (GCol) n = 11, gel seeded with cells (GCell) n = 13 and gel with collagen and cells (GColCell) n = 14. The dashed line represents no change; negative values indicate more compliant gels. Significance value ** *p* = 0.007, Kruskal-Wallis test. (**c**,**d**) Biomechanical maps produced by OptoRheo of MCF-7 clusters expressing tdTomato (shown in purple) encapsulated in hydrogels and (**d**). MCF-7 clusters from the same cell line in hydrogel supplemented with collagen I labelled with Cy5 (shown in green) monitored over three days. Spheres depict microsphere probes (not to scale) assigned a colour to reflect the local stiffness ($G_{0}^{\prime }$). (**e**,**f**) Spatio-temporal changes in $G_{0}^{\prime }$ values with relative distance from the edge of the cell clusters in gel in the absence (**e**) and presence (**f**) of collagen over three days. Reprinted with permission from Ref. [102]. Copyright 2023 Springer Nature.

Another milestone in 2023 was the development of OptoRheo, an instrument that integrated light sheet fluorescence microscopy with particle tracking microrheology. OptoRheo enabled simultaneous imaging and microrheological measurements in live 3D biological systems. Its dual-modality approach allowed researchers to characterize extracellular matrix (ECM) stiffness and viscosity variations in cancer models as seen in Figure 20, offering potential applications in drug delivery optimization and advanced disease modelling. The minimally invasive nature of OptoRheo allowed for extended observation of cellular and ECM dynamics, making it a transformative tool for studying cell–matrix interactions over long timescales [102].

**Figure 21:**
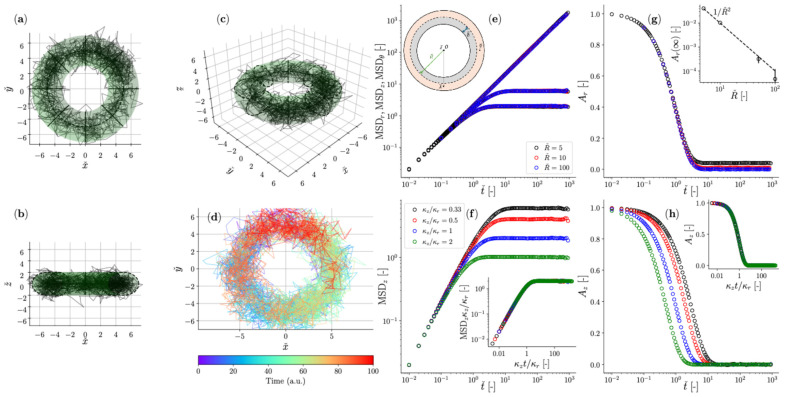
(**a**–**d**) Trajectory of a colloidal particle suspended in a Newtonian fluid and constrained by a toroidal optical trap with major radius $\overset{ˇ}{R}=5$, small radius $\overset{ˇ}{b}=1$, and $\kappa_{z}/\kappa_{r}=\frac{1}{3}$. (**e**–**h**) Similar simulation conditions to (**a**–**d**), but exploring the effects of varying $\overset{ˇ}{R}$ on the MSD in (**e**), and on the normalized position autocorrelation function in the radial $A_{r}\left(t\right)$ in (**g**). The inset in (**g**) shows the steady-state value of $A_{r}\left(t\right)$ as a function of $\overset{ˇ}{R}$. (**f**,**h**) show the effects of varying the ratio $\kappa_{z}/\kappa_{r}$ on the MSD in (**f**), and on the normalized position autocorrelation function $A_{z}\left(t\right)$, both only in the axial direction. The insets in (**f**,**h**) show the master curves for MSD_z_ and $A_{z}\left(t\right)$ when the same data shown in the main are plotted against $\kappa_{z}t/\kappa_{r}$, respectively, and the MSD_z_ is normalized by the variance of the optical trap in the z direction [26]. Reprinted with permission from Ref. [110]. Copyright 2024 MDPI.

Meanwhile, Optical Halo, introduced in 2024, addressed a longstanding challenge in microrheology: measuring materials’ viscoelastic properties at low frequencies. Utilizing a ring-shaped Bessel beam in optical tweezers, Optical Halo enabled continuous particle tracking along azimuthal directions as shown in Figure 21, effectively overcoming the limitations of conventional methods. This advancement allowed for the study of materials with long relaxation times, such as bio-polymers and highly concentrated solutions, and expanded the frequency range accessible to microrheological investigations [110].

**Figure 22:**
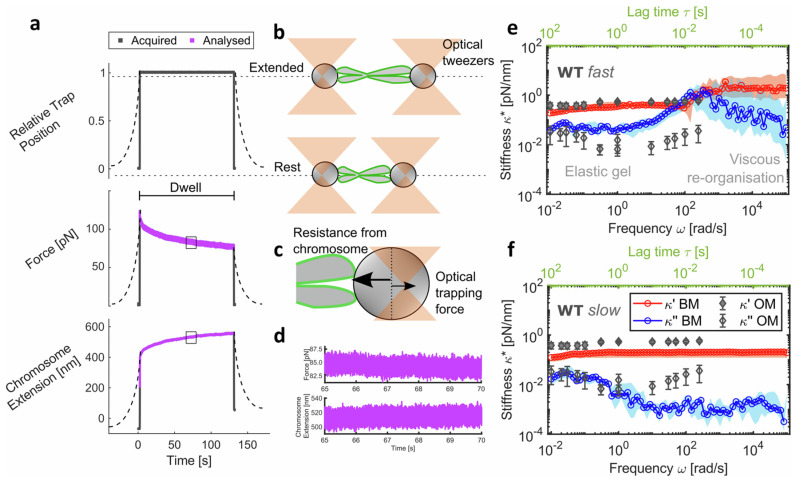
(**a**) Schematic of microrheology experimental procedure. Dashed lines represent data at a force-loading rate of 0.2 μm/s and solid lines for 100 μm/s. Data in purple were analyzed to provide broadband mechanical response. (**b**) Schematic representation of the tweezer and chromosome positions from (**a**). (**c**) Opposing forces experienced at bead handles (one shown) in the non-equilibrium state. (**d**) Zoomed-in sub-region of the analyzed force at one bead and chromosome extension data. (**e**) Complex stiffness $\kappa^{*}\left(\omega \right)$ with frequency (bottom axis in black) and lag time τ (top axis in green) from broadband microrheology (BM) of WT chromosomes at 100 μm/s (median and 95% CI; *n* = 14 chromosomes), highlighting regions of viscous reorganization and gel-like behaviour. Data in blue are the viscous modulus $\kappa^{\prime \prime }\left(\omega \right)$ and in red are the elastic modulus $\kappa^{\prime }\left(\omega \right)$. (**f**) $\kappa^{*}\left(\omega \right)$ at 0.2 μm/s force-loading rate (median and 95% CI; *n* = 15 chromosomes) of WT chromosomes. (e) and (**f**) are both overlaid with oscillatory microrheology (OM) data from Meijering et al. (2022). Schematics shown are not to scale. Data are provided in a Source Data file. Reprinted with permission from Ref. [28]. Copyright 2025 Springer Nature.

The most recent innovation by Mendonca et al. [28] involved the use of optical tweezers in combination with microfluidic channels to investigate, for the first time, the role of the mitotic chromosome periphery in chromosome mechanics. In this study, a novel analytical method was developed for extracting the mechanical properties of single chromosomes from force-extension experiments. The analytical procedure enabled broadband microrheology across seven decades of frequency, reaching an upper frequency limit of nearly 10^5^ rad/s as shown in Figure 22. This work demonstrates the capability of optical tweezers to explore the contributions of other chromosome-related structures to their mechanical behaviour, which are not extensively studied and, in most cases, still completely unexplored in the literature [28].

Collectively, these advancements demonstrate the trajectory of particle tracking microrheology from ecological applications to cutting-edge biomedical research. By integrating innovative technologies and methodologies, microrheology has become an indispensable tool for exploring the physical and mechanical properties of complex systems, paving the way for new insights into both natural ecosystems and cellular dynamics.

## 4. Conclusions

Microrheology has transformed our understanding of the mechanical properties of complex systems at the microscale. By integrating active and passive techniques, it has enabled researchers to study the viscoelastic behaviour of biological systems and soft materials with unprecedented precision, offering insights that were previously unattainable.

In biology, microrheology has revealed how cells interact with and adapt to their mechanical environment. Techniques such as particle tracking and optical tweezers have uncovered the influence of mechanical properties on key processes like migration, division, and mechanotransduction. These findings are particularly significant in understanding diseases like cancer, where the mechanical properties of cells and their microenvironment are closely tied to progression and metastasis.

In materials science, microrheology has been instrumental in studying the dynamics of polymers, gels, and protein solutions. By capturing spatial heterogeneities and mapping phase transitions, it has contributed to the design of materials with tailored mechanical properties, fostering advancements in both scientific research and industrial applications.

Future work in PTM may focus on investigating a wider range of biological structures, potentially in vivo, as well as characterizing novel materials to optimize their functionality. Though challenges remain, particularly in interpreting data from heterogeneous systems, the continued development of microrheological techniques promises to address these complexities. By bridging biology and materials science, microrheology has established itself as a critical tool for modern research, offering a deeper understanding of the mechanical behaviour of complex systems and driving innovation across disciplines.

## Figures and Tables

**Table 1:** Comparison of microrheology techniques (as in Figure 1): advantages and limitations.

Technique	Advantages	Drawbacks/Limitations
PVPTM	Simple equipment; Non-invasive measurements	Limited frequency range; Not applicable to stiff or non-equilibrium systems
MT	Suitable for high-viscoelasticity fluids; Biologically safe magnetic control	Sophisticated and not easily multiplexed
OT	Extremely sensitive; Useful for broadband rheology	Sophisticated and expensive instrumentation
DLS	Commercially available equipment; Suitable for dilute samples	Cannot resolve spatial heterogeneity; Not suitable for highly turbid samples
DWS	Can probe opaque/turbid samples; Excellent performance in highly turbid samples	Requires multiple scattering; Complex data analysis; Cannot resolve spatial heterogeneity
AFM	High spatial resolution; Suitable for tiny sample volumes	Limited to surface contact measurements; Low throughput

## Data Availability

No new data were created or analyzed in this study. Data sharing is not applicable to this article.
